# The Comprehensive Autistic Trait Inventory (CATI): development and validation of a new measure of autistic traits in the general population

**DOI:** 10.1186/s13229-021-00445-7

**Published:** 2021-05-17

**Authors:** Michael C. W. English, Gilles E. Gignac, Troy A. W. Visser, Andrew J. O. Whitehouse, James T. Enns, Murray T. Maybery

**Affiliations:** 1 School of Psychological Science, University of Western Australia, Perth, WA Australia; 2 Telethon Kids Institute, Perth, WA Australia; 3 Department of Psychology, University of British Columbia, Vancouver, BC Canada

**Keywords:** Autism, Autistic traits, Broader autism spectrum, Self-report, Questionnaire, Factor analysis, Subscales, General population

## Abstract

**Background:**

Traits and characteristics qualitatively similar to those seen in diagnosed autism spectrum disorder can be found to varying degrees in the general population. To measure these traits and facilitate their use in autism research, several questionnaires have been developed that provide broad measures of autistic traits [e.g. Autism-Spectrum Quotient (AQ), Broad Autism Phenotype Questionnaire (BAPQ)]. However, since their development, our understanding of autism has grown considerably, and it is arguable that existing measures do not provide an ideal representation of the trait dimensions currently associated with autism. Our aim was to create a new measure of autistic traits that reflects our current understanding of autism, the Comprehensive Autism Trait Inventory (CATI).

**Methods:**

In Study 1, 107 pilot items were administered to 1119 individuals in the general population and exploratory factor analysis of responses used to create the 42-item CATI comprising six subscales: *Social Interactions*, *Communication*, *Social Camouflage*, *Repetitive Behaviours*, *Cognitive Rigidity*, and *Sensory Sensitivity*. In Study 2, the CATI was administered to 1068 new individuals and confirmatory factor analysis used to verify the factor structure. The AQ and BAPQ were administered to validate the CATI, and additional autistic participants were recruited to compare the predictive ability of the measures. In Study 3, to validate the CATI subscales, the CATI was administered to 195 new individuals along with existing valid measures qualitatively similar to each CATI subscale.

**Results:**

The CATI showed convergent validity at both the total-scale (*r* ≥ .79) and subscale level (*r* ≥ .68). The CATI also showed superior internal reliability for total-scale scores (*α* = .95) relative to the AQ (*α* = .90) and BAPQ (*α* = .94), consistently high reliability for subscales (*α* > .81), greater predictive ability for classifying autism (Youden’s Index = .62 vs .56–.59), and demonstrated measurement invariance for sex.

**Limitations:**

Analyses of predictive ability for classifying autism depended upon self-reported diagnosis or identification of autism. The autistic sample was not large enough to test measurement invariance of autism diagnosis.

**Conclusions:**

The CATI is a reliable and economical new measure that provides observations across a wide range of trait dimensions associated with autism, potentially precluding the need to administer multiple measures, and to our knowledge, the CATI is also the first broad measure of autistic traits to have dedicated subscales for social camouflage and sensory sensitivity.

**Supplementary Information:**

The online version contains supplementary material available at 10.1186/s13229-021-00445-7.

## Introduction

### Autism and autistic traits

It is well-established that core behaviour characteristics of autism spectrum disorder (hereafter, autism) vary in both severity and scope. In addition to clinically defined autism, it is also becoming increasingly recognised that many individuals who do not meet the diagnostic criteria display traits and behaviours qualitatively similar to the symptoms of the condition. These ‘sub-threshold’ autistic traits were first noted in the parents and close relatives of autistic children [1], but are now known to be normally distributed across the general population more broadly [2–4], suggesting that the construct of autism may be trait-like, forming a continuum in the general population. Consistent with a continuum, the heritability of autism calculated in twin studies is estimated to be 70–81% [5, 6], while the heritability of autistic traits in the general population is similarly high (62–76%) [7]. More striking evidence of continuity is that overlapping genetic aetiology has been demonstrated for ASD and autistic traits in the general population in large-scale studies using very different methods: twin concordance [8], common genetic variants [9, 10] and de novo variants [10].

To better understand the distribution and influence of autistic traits in the general population, several authors have developed psychometric scales that measure the prevalence of associated behaviours. The Autism-Spectrum Quotient (AQ) [2], a 50-item self-report measure that assesses a variety of trait dimensions associated with autism, has been extensively used in autism research since its publication in 2001, recording 2840 citations in Scopus at the time of writing. Alternative measures often used to quantify autistic traits for research purposes include the Social Responsiveness Scale (SRS-2) [11] and the Broad Autism Phenotype Questionnaire (BAPQ) [12], with 823 and 254 Scopus citations, respectively.

Due to the qualitative similarities found between individuals with clinical and ‘subthreshold’ trait autism, measures of autistic traits have often been used to facilitate autism research by recruiting non-autistic individuals who vary in levels of self-reported autistic traits. Studying autistic-trait groups has several benefits in enabling solutions to methodological challenges that are often present in investigating clinical autism, such as in recruiting a sufficient sample size, controlling for the substantial comorbidity of other conditions associated with autism, and managing the complexity of forming appropriately matched comparison groups [13, 14]. Furthermore, behavioural differences reported between individuals with low and high levels of autistic traits often mirror differences observed been clinically diagnosed autistic and non-autistic individuals [15].

An additional strength of these scales is their ability to not only provide an overall assessment of autistic trait levels by means of a total-scale score, but to also assess trait dimensions separately. For example, the AQ was designed to measure five subscales: *Social Skill*, *Attention Switching*, *Attention to Detail*, *Communication*, and *Imagination,* while the BAPQ has three subscales: *Aloof Personality*, *Pragmatic Language*, and *Rigid Personality*. This multi-dimensional approach to the assessment of autistic traits has been crucial in identifying that certain characteristics relate more strongly to some dimensions of autism than others. For example, superior figure disembedding has been associated with greater social difficulties, as assessed by either the AQ [16] or the BAPQ [17], but not with any of the other trait dimensions assessed by these two questionnaires. Similarly, variation in adaptation effects following exposure to asynchronous multisensory stimuli is associated with differing levels of attention to detail on the AQ, while the other autistic trait dimensions showed no such associations [18]. Given the heterogeneous nature of autism, it is important to investigate the distinct as well as shared characteristics of specific dimensions of autism, and not take a ‘broad brush’ approach that treats all autistic individuals as a homogenous group.

### Limitations of existing measures

As our understanding of autism has continually evolved and developed, it has become increasingly apparent that there are gaps in the existing broad measures of autistic traits. Critically, no single measure provides an adequate assessment of all the main dimensions currently associated with autism. For example, sensory sensitivity was added to the diagnostic criteria for autism spectrum disorder in the DSM-5 [19] but barely features across the AQ, BAPQ, or SRS-2. Similarly, physical repetitive behaviours are largely absent from these scales as well. Though researchers have developed specific questionnaires to fill such gaps (e.g. Adult Repetitive Behaviour Inventory [20] and Glasgow Sensory Questionnaire [21]), investigating these features presents additional challenges in combining data across measures with different psychometric properties.

Furthermore, total-scale scores for the AQ, BAPQ, and SRS-2 are often interpreted to be broad, general representations of autistic trait levels across individuals, but it is questionable how representative a single total-scale score can be when certain known trait dimensions are under-represented within the scales. Compounding this issue is evidence that certain trait dimensions are, at best, relatively weakly correlated [22–28], mimicking the heterogenous nature of autism. This means that elevated scores on one dimension cannot be used to infer similar levels on other dimensions, and further brings into question the interpretability of total-scale scores based on a subset of known trait dimensions (for a demonstration, Fig. 2 of English et al. [22] shows the subscale variability of 49 individuals with identical total-scale AQ scores). With these concerns in mind, ideally, in investigating comprehensive psychometric measures of trait autism, researchers should evaluate the reliability of the total score as derived from subscale scores rather than from item scores as is typically used in calculation of Cronbach alpha [29].

### Overview

Currently, there is no single, comprehensive option for researchers aiming to capture the broad range of trait dimensions associated with autism or derive a valid overall measure of autistic traits. To address this issue, we aimed to create a new scale, the Comprehensive Autism Trait Inventory (CATI), a scale that maintains the strengths of existing scales, like the AQ and BAPQ, but also serves to fill some crucial gaps in their assessments of the various autistic trait dimensions. In this paper, we outline the development and assessment of the CATI across three studies. In Study 1, we describe the development of 107 pilot items and, following administration of the pilot version of the CATI to a large sample of adult participants, the results of an exploratory factor analysis that reduced the number of items to 42, spread across six trait dimensions. In Study 2, we recruited a second large sample to which the final version of the CATI was administered, using the collected data to support the six-factor structure of the CATI with confirmatory factor analysis. We also compare the CATI’s ability to classify and discriminate autistic and non-autistic individuals to that of the AQ and BAPQ using logistic regression. Finally, in Study 3, we establish convergent validity for each of the CATI’s six subscales by administering the CATI to a third adult sample alongside several established scales that each measure a trait dimension qualitatively similar to that measured by a subscale in the CATI.

## Study 1: CATI scale development

The aims of Study 1 were threefold: (1) the development of a large set of pilot items; (2) administration of these items to a large sample of participants to identify a psychometrically supported factor structure via exploratory factor analysis; and (3) reducing the number of items to a reasonable number for the final version of the scale while maintaining strong psychometric properties.

### Methods

#### Ethical approval

Approval to conduct all studies was received from the Human Research Ethics Office at the University of Western Australia (reference RA/4/20/5546) and the research was carried out in accordance with the provisions of the World Medical Association Declaration of Helsinki. As the research was conducted entirely online, all participants were presented with an online, downloadable version of an approved information sheet and consent form that required endorsement prior to any experimental participation. Following the completion of the study, participants were also presented with a downloadable debrief form outlining the purpose of the study in added detail. Participants received a £1.66 reimbursement for their participation, with most participants completing the study within 20 min.

#### Participants

Participants were recruited through Prolific Academic, an online portal that enables individuals from the general population to complete online-based studies and receive a monetary reimbursement in return. The recruitment strategy was generally identical for the three samples and involved targeting individuals from five English-speaking countries (UK, USA, Canada, Australia, and New Zealand) and recruiting a relatively even proportion of male and female participants. Given the aim of developing a questionnaire that can assess autistic traits across the entire autism continuum, participants who identified as autistic (regardless of whether a formal diagnosis had been received or not) were retained in the sample.

A total of 1256 individuals were recruited into Study 1. Prior to any analyses, participants were removed from the dataset if one or more of the following conditions were observed: (a) self-reported a primary language other than English (*n* = 5); (b) failed any of the attention checks built-in to the questionnaire (*n* = 83); or (c) completed the questionnaires too quickly (i.e., < 5 min; *n* = 3). Of the remaining 1166 participants, there was a relatively even male-to-female ratio of participants (569 male, 581 female, 16 sex not given). Participants ranged from 18 to 82 years in age (*M* = 37.33, SD = 12.85). Within the sample, 17 participants (10 male, 6 female, 1 sex not given) reported having previously received a formal diagnosis of ASD. The proportion of diagnosed autistic participants in the sample (1.46%) approximates current USA population estimates (1 in 59, or 1.69% [30]). A further 30 participants (14 male, 16 female) reported that they self-identified as autistic but had not received a formal diagnosis. The remaining 1119 participants (545 male, 559 female, 15 sex not given) did not report having received a diagnosis of autism or otherwise self-identifying as autistic (i.e. non-autistic). Additional participant details can be found in Additional file 1: Table S1.

### Materials

#### Pilot version of the Comprehensive Autism Trait Inventory (CATI)

The pilot version of the CATI was developed in conjunction with input from the authors, autism researchers, clinicians who are personal contacts of the authors, and a focus group of autistic students studying at the University of Western Australia. Six “themes” were initially suggested that would reflect potential subscales and focus item development, including social preferences (motivation), communication ability (verbal and non-verbal), repetitive movements, atypical sensory sensitivity, monotropic mindset (i.e. restricted interests, insistence on sameness), and rigidity/inflexibility. These themes were designed to relate to diagnostic criteria for autism but reflect qualitatively similar behaviours and characteristics seen in non-autistic individuals from the general population. Feedback led to the inclusion of additional items specific to social skills which were distinct from social preferences. Considerable interest in the measurement of traits represented more strongly in women led to the inclusion of items relating to social camouflage [31–34]. While not directly referenced in the diagnostic criteria for autism, we suggest that camouflaging items should be considered because (1) camouflaging was reported to be a major feature of their condition by autistic adults in our focus group, (2) inclusion of a camouflaging subscale may increase the sensitivity of the total scale in identifying autistic traits in women, and (3) the camouflaging items could be dropped from the final questionnaire if psychometric analyses did not support keeping them.

Multiple rounds of feedback helped to develop additional items, adjust the phrasing of others, and remove redundant items. The process resulted in in 107 items, spread across the six themes described above plus the additional themes of social skills and social camouflaging. Each item took the form of a statement that could be responded to using a five-point Likert scale that included the responses: “Definitely disagree”, “Somewhat disagree”, “Neither agree nor disagree”, “Somewhat agree”, and “Definitely agree”. Items were phrased such that there was a mixture of positively keyed and negatively keyed items, with about a quarter of the items negatively keyed. For this version of the CATI, item presentation order was randomised for each participant.

#### Procedure

The study was presented using the Qualtrics survey platform. First, participants voluntarily entered some basic demographics information, which included age, sex, and English-language proficiency. Participants then completed the 107-item pilot version of the CATI. For this version of the questionnaire, participants were required to make a response to each item on each page before they could proceed to the next page, thus ensuring there was no missing questionnaire data. To help identify participants who were not following instructions or reading the statements carefully, three “attention check” items directing participants to select a particular response (e.g. “For this item, please select ‘Somewhat disagree’”) were inter-mixed with the pilot items with their positions randomised for each participant. Finally, participants were asked whether they had either previously received a formal diagnosis of autism or whether they otherwise identified as being autistic.

### Statistical analyses

All analyses were conducted using R [3.6.2] and RStudio [1.2.5033]. Exploratory factor analyses (EFA), using the Psych package [1.9.12.31], were conducted on the scores obtained using the pilot version of the CATI to derive a valid factor structure and select items for the final version of the scale. The number of factors to be extracted was determined based on the results of parallel analysis, and the general interpretability of the resulting item loadings across the extracted factors. Polychoric correlations were used to estimate associations between questionnaire items and the analysis was conducted using weighted least squares estimation and an oblimin rotation method. Items required a loading > .30 to be interpreted as part of a factor. After selecting the items to be retained for the final version of the CATI (see below), these candidate items were subject to a second EFA using the same parameters to ensure that the factor structure had not changed substantially following the removal of the unselected items. At this point, internal consistency was estimated for the subscale scores with Cronbach alpha,^1^ and total-scale score using McDonalds omega hierarchical [29], with values of .80 or greater expected for research purposes [35].

### Item selection guiding principles

Following an EFA of scores obtained on the 107-item pilot version of the CATI a subset of items was selected into the final version of the scale. To ensure adequate consistency within factors, seven items were retained for each identified factor, with the item selection following several guiding principles. Item selection began by considering the strongest loading items on each factor, except when these items loaded substantially on another factor, did not appear to discriminate between autistic and non-autistic participants, or were deemed to have problematic phrasing that was not identified during the initial development of the CATI. Finally, item selection for the potential *Sensory Sensitivity* factor was modified to allow for at least one item from each of the five primary sensory domains to be included in the final scale.

### Results

#### Identifying the factor structure of the CATI and selection of items

Participant responses were first screened for acceptable levels of quality and those who did not meet certain criteria were removed (see the "Participants" section). A principal components parallel analysis was conducted to help determine the upper limit of the number of components that might be extracted using exploratory factor analysis. While parallel analysis suggested that 10 components were identifiable in the data, attempts to extract 7–10 factors resulted in the creation of factors with only 2–5 items and low internal consistency reliability for the corresponding scales. Ultimately, a six-factor solution that accounted for 45% of the variance was selected, as it was the most interpretable. The pattern matrix for the exploratory factor analysis on the 107 pilot items is reported in Additional file 1: Table S2. From the eight different dimensions that guided initial item development, social skills and social preferences were merged into a single *Social Interactions* factor, and the monotropic mindset dimension was dropped, as many of its items tended to load on other factors (i.e. *Cognitive Rigidity* or *Repetitive Behaviours*).

Seven items were selected from each of the six factors following the principles outlined earlier, which resulted in a total of 42 selected items.^2^ A second parallel analysis was conducted on the 42 items, and six components were identified. A second exploratory factor analysis was conducted on these 42 items to verify that the item loadings were consistent with the previous analysis. The model now explained 55% of the variance, and the pattern matrix for this analysis is reported in Table 1. Internal consistency was calculated for each of the subscales, with Cronbach alpha coefficients in the range of .80–.93, and total-scale internal consistency (stratified Cronbach alpha) of .97 (reported in detail in Table 1). As total-scale internal consistency may be over-estimated for multi-dimensional scales using Cronbach alpha, McDonald’s omega hierarchical (an alternative index based on inter-subscale correlations instead of inter-item correlations) was also calculated [29], and showed an acceptable level of consistency (.83). All subscales were found to inter-correlate positively, with correlations ranging in size from *r* = .33 to .56. The strong item loadings, good internal consistency, and clear interpretability of the factor structure supported using the 42 selected items for the final version of the CATI.

**Table 1:** Pattern matrix for Study 1 following exploratory factor analysis for the 42 items selected to be retained for the final version of the CATI, together with internal consistency for each of the subscales (Cronbach alpha), total scale (Cronbach alpha stratified and McDonald’s omega hierarchical), and bifactors identified in Study 2 (Cronbach alpha)

Factor/item	SOC	COM	REP	CAM	RIG	SEN
Social interactions [SOC] (*α* = .93)						
Social interaction is easy for me*	**.89**	.08	.01	− .01	.01	− .03
I generally enjoy social events*	**.89**	.02	− .03	− .19	.00	.09
I find social interactions stressful	**.82**	− .05	.05	.09	− .02	.11
Social occasions are often challenging for me	**.80**	.02	− .01	.11	.03	.09
I am confident and capable when meeting new people*	**.78**	.06	.05	.01	.02	− .05
In social situations, I try to avoid interactions with other people	**.74**	.06	.10	.09	.01	− .01
I find it difficult to make new friends	**.72**	.12	− .05	.09	.03	− .04
Communication [COM] (*α* = .83)						
I can tell how people feel from their facial expressions*	.05	**.82**	− .01	− .20	.00	− .03
Reading non-verbal cues (e.g. facial expressions, body language) is difficult for me	.02	**.82**	.07	.04	− .02	.01
I find it easy to sense what someone else is feeling*	.11	**.75**	− .07	− .12	.05	− .09
Metaphors or ‘figures of speech’ often confuse me	− .17	**.57**	.10	.09	.00	.19
I rarely use non-verbal cues in my interactions with others	− .05	**.57**	− .01	.01	.09	.01
I have difficulty understanding the 'unspoken rules' of social situations	.22	**.54**	.06	.15	− .04	.08
I have difficulty understanding someone else’s point-of-view	.04	**.54**	.05	.04	.05	.12
Repetitive behaviour [REP] (*α* = .85)						
I often find myself fiddling or playing repetitively with objects (e.g. clicking pens)	.07	− .06	**.83**	.00	− .03	− .02
There are certain objects that I fiddle or play with that can help me calm down or collect my thoughts	− .02	.06	**.70**	− .06	.04	.10
I often rock when sitting in a chair	− .03	.05	**.65**	.04	− .04	.01
There are certain repetitive actions that others consider to be 'characteristic' of me (e.g. stroking my hair)	− .07	.03	**.64**	.09	.09	− .03
I have a tendency to pace or move around in a repetitive path	.03	.10	**.61**	.04	.04	.06
I engage in certain repetitive actions when I feel stressed	.08	.00	**.60**	.04	.17	.03
I have certain habits that I find difficult to stop (e.g. biting/tearing nails, pulling strands of hair)	.10	− .01	**.58**	− .01	.04	− .04
Social camouflage [CAM] (*α* = .86)						
Sometimes I watch people interacting and try to copy them when I need to socialise	.03	.13	.03	**.70**	.01	.00
I look for strategies and ways to appear more sociable	− .06	− .02	.04	**.70**	.08	− .06
Before engaging in a social situation, I will create a script to follow where possible	.11	.16	− .01	**.65**	.01	.00
I rely on a set of scripts when I talk with people	.07	.26	− .05	**.64**	.00	.01
I try to follow certain 'rules' in order to get by in social situations	− .03	.08	− .15	**.62**	.26	.02
I expend a lot of mental energy trying to fit in with others	.23	− .05	.08	**.61**	− .06	.04
When interacting with other people, I spend a lot of effort monitoring how I am coming across	.14	− .17	.12	**.59**	.06	.05
Cognitive rigidity [RIG] (*α* = .81)						
I like to stick to certain routines for every-day tasks	.18	− .01	− .14	.04	**.73**	− .03
I like my belongings to be sorted in certain ways and will spend time making sure they are that way	− .10	.02	.02	.03	**.70**	.03
There are certain activities that I always choose to do the same way, every time	.18	− .03	.02	− .04	**.70**	− .11
I often insist on doing things in a certain way, or re-doing things until they are ‘just right’	− .03	.04	.11	− .02	**.63**	.05
I feel discomfort when prevented from completing a particular routine	.11	.01	.04	.03	**.57**	.15
I like to arrange items in rows or patterns	− .11	.08	.15	.06	**.52**	.08
It annoys me when plans I have made are changed	.23	− .08	.05	− .02	**.40**	.09
Sensory sensitivity [SEN] (*α* = .80)						
I would describe myself as a very sensory-sensitive person	− .03	− .08	− .01	.01	.13	**.66**
I am sensitive to flickering lights	.12	− .02	− .03	.02	− .03	**.62**
I react poorly to unexpected loud noises	.18	.03	− .05	− .04	.09	**.59**
There are times when I feel that my senses are overloaded	.19	− .06	.15	.00	.01	**.57**
I am over-sensitive to touch	.17	.12	.03	.10	.00	**.53**
I am over-sensitive to particular tastes (e.g. salty, sour, spicy, or sweet)	− .06	.22	.05	.03	.00	**.52**
Sometimes the presence of a smell makes it hard for me to focus on anything else	− .04	.07	.08	− .02	.08	**.49**
Total scale (*α* = .97^a^, ω^h^ = .83^b^)						
Social bifactor [SOC, COM, CAM] (*α* = .92)						
Non-social bifactor [REP, RIG, SEN] (*α* = .90)						

*N* = 1166. Loadings > .30 in bold text. *Negatively keyed items

^a^Cronbach alpha stratified across subscales [36]

^b^McDonald’s omega hierarchical estimated as outlined in Gignac [29]

## Summary

In Study 1, we generated 107 pilot items centred around eight different themes that were administered to a large sample of adults to enable an exploratory factor analysis. The results of the EFA suggested that there were six factors that could be well-defined by the items that covered *Social Interactions*, *Communication*, *Sensory Sensitivity*, *Repetitive Behaviour*, *Cognitive Rigidity*, and *Social Camouflage*. Items from two of the initial themes, social preferences and social skill, clustered together to form the single *Social Interactions* factor, and items from the monotropic mindset theme were largely distributed across the *Repetitive Behaviour* and *Cognitive Rigidity* themes, rather than forming a factor of their own. All things considered, 42 items were selected to create the final version of the CATI, with seven items covering each of the six identified subscales. For the final item set, internal consistency was high for the six subscales and the total scale.

## Study 2: CATI factor structure verification

Having created the 42-item CATI in Study 1, we next performed several confirmatory factor analyses on various configurations of the six-factor solution identified in Study 1 (i.e. correlated factors, bifactor, higher-order factor) using a new sample of participants to determine the best overall structure of the CATI (see “Statistical analyses” section for detailed descriptions of the models tested). A further aim was to examine how the CATI compared with two contemporaries, the AQ and BAPQ, in terms of ability to discriminate and classify autistic and non-autistic individuals using logistic regression. The AQ and BAPQ were chosen as both are considered broad measures of autistic traits and, like the CATI, are not restricted to a specific trait dimension.

### Methods

#### Participants

A large sample of adults (*n* = 1145) who had not previously taken part in Study 1 were recruited into Study 2. As with Study 1, prior to any analysis, participants were removed from the dataset if one or more of the following conditions were observed: (a) self-reported a primary language other than English (*n* = 2); (b) failed any of the attention checks built-in to the questionnaire (*n* = 5); or (c) completed the questionnaires too quickly (i.e., < 5 min; *n* = 13).^3^ Of the remaining 1119 participants, there was a relatively even male-to-female ratio of participants (557 male, 552 female, 10 sex not given), similar to Study 1. Participants ages ranged from 18 to 75 years (*M* = 37.41, SD = 12.59). Within the sample, 9 participants (3 male, 5 female, 1 sex not given) reported having previously received a formal diagnosis of ASD. The proportion of diagnosed autistic participants in the sample (.80%) is slightly below current USA population estimates (1 in 59, or 1.69% [30]). A further 42 participants (24 male, 18 female) reported that they self-identified as autistic but had not received a formal diagnosis. The remaining 1068 participants (530 male, 529 female, 9 sex not given) did not report having received a diagnosis of autism or otherwise self-identifying as autistic (i.e. non-autistic).

### Materials

#### Comprehensive Autism Trait Inventory (CATI)

Readers seeking to access the CATI can find the full scale and scoring key in Additional file 2 of this article. Following an exploratory factor analysis of the pilot version in Study 1, 42 items were chosen for the final version with seven items associated with each of the subscales, *Social Interactions*, *Sensory Sensitivity*, *Repetitive Behaviours*, *Communication Difficulty*, *Cognitive Rigidity*, and *Social Camouflaging*. The final version used the same five-point Likert response scale as was used for the pilot version. Unlike the pilot version, the final version of the CATI has a fixed item order with items from different subscales and negatively keyed items spread relatively evenly throughout the scale.

#### Autism Spectrum Quotient (AQ)

The AQ [2] is a 50-item self-report questionnaire designed to quantify autistic traits in neurotypical individuals. Items are in the format of statements such as “*I enjoy social chit-chat*” and are responded to using a four-point Likert scale: “Definitely agree”, “Slightly agree”, “Slightly disagree”, and “Definitely disagree”. Approximately half of the items are negatively keyed. Scoring was originally dichotomous (i.e. 0–1), with responses collapsed into “agreement” and “disagreement” (scored ‘1’ and ‘0’ respectively), without regard to strength of item endorsement. But beginning with Austin (2005), others have scored the AQ using the full possible range (i.e. 1–4), which has been found to improve item discriminability [37]. This alternative scoring format is used in the current study. The original authors assigned ten items to five separate trait dimensions labelled *Social Skill*, *Attention Switching*, *Attention to Detail*, *Communication*, and *Imagination*. Although recent evidence indicates that alternative factor structures show more psychometric support [22], the original subscales are still used widely and thus were examined in the present study.

#### Broad Autism Phenotype Questionnaire (BAPQ)

The BAPQ is a 36-item self-report questionnaire originally designed to measure autistic traits in the close relatives of autistic individuals [12], but commonly used more broadly across the general population as well. Items take the form of statements (e.g. “*I like being around other people*”) that are responded to using a six-point Likert scale: “Very rarely”, “Rarely”, “Occasionally”, “Somewhat Often”, “Often”, and “Very often”. Approximately half of the items are negatively keyed. Responses are scored 1–6, with higher scores representing greater endorsement of autistic traits. Items are evenly distributed across three subscales, *Pragmatic Language*, *Aloof Personality*, and *Rigid Personality*, with later studies largely supporting this factor structure [38, 39].

#### Procedure

The procedure was largely identical to that used for Study 1. However, instead of the 107 pilot CATI items being administered to participants in a random order, the AQ, BAPQ, and final version of the CATI were administered in random order to participants. Furthermore, one attention-check item, similar to those randomly interspersed in the pilot CATI in Study 1, was placed at the mid-point of each of the three questionnaires with the item phrased to fit each questionnaire’s statement and response format. All other aspects of the procedure were identical to that used for Study 1.

### Statistical analyses

**Fig. 1:**
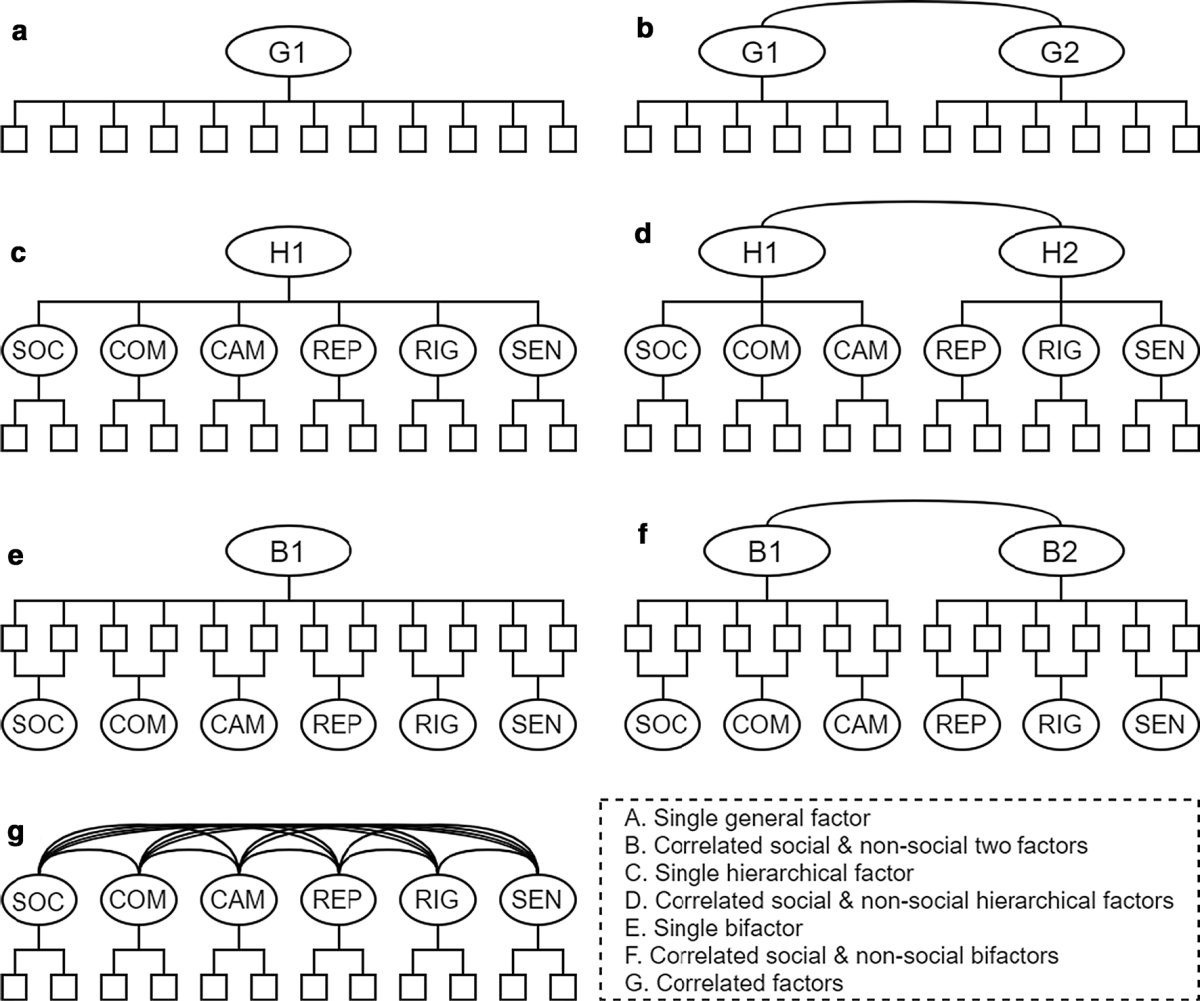
Models assessed using confirmatory factor analysis in Study 2. Squares represent the (observed) items and ellipses represent the latent variables (note that only two of the six items for each lower-order factor are represented, and that item uniqueness is omitted, for clarity and space). *SOC* social interactions, *COM* communication, *CAM* social camouflage, *REP* repetitive behaviours, *RIG* cognitive rigidity, *SEN* sensory sensitivity

All analyses were conducted using R [3.6.2] and RStudio [1.2.5033]. Confirmatory factor analyses (CFA) were conducted using the ‘lavaan’ package [.6–5] (polychoric correlations and weighted least squares estimation). Several model variants of the six-factor structure identified in Study 1 were tested (see Fig. 1 for details). In addition to a correlated factors model, we also tested a model where the six factors were themselves part of a higher-order factor, a model where the six factors existed in tandem with a bifactor, and another model with the six factors replaced by a single general (or common) factor. Further models were inspired from the Diagnostic and Statistics Manual’s [19] separation of two distinct criteria for autism diagnosis—difficulties in social communication and social interaction and restricted/repetitive behaviours and interests—and from previous psychometric analysis of the AQ that has found evidence of a higher-order social factor that subsumes several socially related subscales [40]. These additional model variants separately grouped the social (*Social Interactions*, *Communication*, *Social Camouflage*) and non-social (*Repetitive Behaviour*, *Cognitive Rigidity*, *Sensory Sensitivity*) factors at the second-order level. Model-fit was assessed with the following robust close-fit indices: RMSEA and SRMR (< .08 indicative of fair fit and < .06 indicative of good fit) and CFI and TLI (> .90 indicative of fair fit and > .95 indicative of good fit). The overly powerful chi-square test statistic is also reported for completeness. When comparing alternative models, a TLI increase of .01 was used to indicate practical improvement [41]. Internal consistency was estimated for the subscale scores with Cronbach alpha, with values of .80 or greater expected for research purposes [35]. To determine if male and female participants’ responses on the CATI were similarly structured, measurement invariance analyses were also conducted.

Logistic regression analyses were conducted to determine the CATI’s capacity to classify autistic and non-autistic participants, as well as to provide comparisons with similar analyses conducted using responses to the AQ and BAPQ. To do this, separate regression analyses for each questionnaire were conducted with all of the subscales of each questionnaire entered directly into the model. Each of the three models was assessed by examining Nagelkerke’s pseudo *R*^2^ for the overall model and the individual standardised coefficients for each subscale entered into the model. Further comparisons in predictive capacity were made by conducting hierarchical logistic regression analyses in which the CATI subscales were entered in step 1 and either the AQ or BAPQ subscale in step 2 (and vice versa), to examine the amount of additional unique variance (change in Nagelkerke *R*^2^) accounted for above and beyond the proportion of variance explained by step 1.

### Results

#### Verifying the factor structure of the CATI

As for Study 1, participant responses were screened for acceptable levels of quality and those that did not meet criteria were removed (criteria and numbers reported earlier in the Participants section) prior to any data analysis being conducted. Next, a series of confirmatory factor analyses (CFA) were performed testing various models. The results, which are summarised in Table 2, identified several models that displayed acceptable fit indices, while the models that included a general (common) factor were notably inferior to the rest. Overall, the results indicated that a model that included two correlated bifactors (separate social and non-social bifactors as described in the Methods) was the best fit for the data, *χ*^2^ = 3172.37, *p* < .001, RMSEA = .053, SRMR = .054, CFI = .955, and TLI = .950. While the TLI improvement from the second-best model (single bifactor) was .008, slightly below the threshold of .01 for practical improvement, we suggest that the added usefulness of a model that could be scored on separate social and non-social bifactors in addition to the six subscales is worth the slight increase in model complexity.

**Table 2:** Robust fit indices obtained from confirmatory factor analyses of the data obtained in Study 2, and identical analyses for verification purposes using the data previously obtained in Study 1

	*df*	Confirmatory sample from Study 2					Exploratory sample from Study 1				
		*χ*^2^	CFI	TLI	RMSEA	SRMR	*χ*^2^	CFI	TLI	RMSEA	SRMR
Simple correlated factors	804	5350.41	.915	.909	.071	.065	4535.54	.926	.921	.063	.057
One general factor	819	18,006.04	.679	.662	.137	.134	15,500.19	.708	.693	.124	.114
Two general factors: social and non-social	818	12,440.67	.783	.771	.113	.112	10,391.56	.810	.800	.100	.094
Hierarchical: single	813	5413.77	.914	.909	.071	.073	4961.78	.918	.913	.066	.065
Hierarchical: social and non-social	812	5091.82	.920	.915	.069	.069	4356.95	.930	.925	.061	.059
Bifactor: single	777	3582.16	.948	.942	.057	.058	3842.33	.939	.932	.058	.056
Bifactor: social and non-social	**776**	**3172.37**	**.955**	**.950**	**.053**	**.054**	**31,843.67**	**.952**	**.947**	**.052**	**.050**

The model with the best fit indices is highlighted in bold text

All chi-square tests significant: *p* < .001. *CFI* comparative fit index, *TLI* Tucker–Lewis Index, *RMSEA* root mean square error of approximation, *SRMR* standardised root-mean-square residual

To provide further verification of the model fit, identical analyses were performed on the final item set from Study 1. The results were highly similar, with the dual bifactor model again returning the best fit (see Table 2). A small, but notable, difference was that the TLI difference between the single- and dual-bifactor models was .013 in this dataset, above the .01 threshold previously identified to indicate practical improvement.

#### Correlations between CATI subscales

Pearson correlations were used to examine the relationships between CATI subscale scores and the results are outlined in Table 3. While all of the subscales were inter-correlated positively, there was substantial variation in the magnitude of the correlations. For example, *Social Interactions* and *Social Camouflage* showed a strong correlation, *r* = .549, while *Social Interactions* and *Repetitive Behaviours* showed a smaller correlation, *r* = .294, suggesting that the associations between trait dimensions is not uniform. Additionally, the correlation between the social and non-social bifactors was also statistically significant, *r*(1066) = .58, *p* < .001.

**Table 3:** Pearson correlations between CATI subscales

	Social interactions	Sensory sensitivity	Repetitive behaviours	Communication	Cognitive rigidity
Sensory sensitivity	.350	–			
Repetitive behaviours	.294	.448	–		
Communication	.427	.369	.401	–	
Cognitive rigidity	.256	.435	.482	.360	–
Social camouflage	.549	.444	.514	.462	.417

All correlations were statistically significant, *p* < .001

#### Internal consistency

Internal consistency for each subscale was excellent, with Cronbach alpha ranging from .81 to .94 (see Table 4). These values are similar to what was found in Study 1 and satisfy the recommended threshold for basic research [35]. Additionally, these values are either comparable or exceed similar levels of consistency found for the AQ and BAP in our sample (see Table 4 for individual values). Internal consistency for the total-scale CATI score was again excellent, as assessed by McDonald’s omega hierarchical and also stratified Cronbach alpha, and exceeded levels observed for the AQ and BAPQ (see Table 4).

**Table 4:** Internal consistency for the CATI, AQ, and BAPQ, assessed using Cronbach alpha for the subscales (and CATI bifactors), and McDonald’s omega hierarchical and stratified Cronbach alpha for the total-scale scores

Sub-scale		Cronbach Alpha
CATI		
Social interactions		.94
Sensory sensitivity		.83
Repetitive behaviours		.84
Communication		.83
Cognitive rigidity		.81
Social camouflage		.84
Social bifactor [social interactions, communication, social camouflage]		.91
Non-social bifactor [sensory sensitivity, repetitive behaviour, cognitive rigidity]		.89
AQ		
Social skill		.85
Attention switching		.74
Attention to detail		.71
Communication		.73
Imagination		.69
BAPQ		
Aloof personality		.93
Pragmatic language		.82
Rigid personality		.88

Total-scale	McDonald’s Omega hierarchical^b^	Stratified Cronbach alpha^a^
CATI	.81	.95
AQ	.71	.90
BAPQ	.76	.94

^a^Cronbach alpha stratified across subscales [36]

^b^McDonald’s omega hierarchical estimated as outlined in Gignac [29]

#### Precision across total-scale scores

**Fig. 2:**
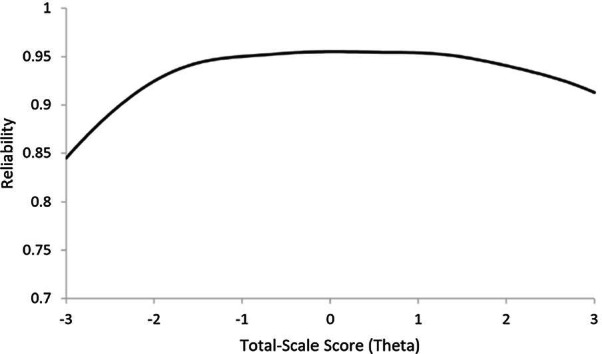
Total information curve (transformed to reliability) for the CATI total-scale. X-axis represents measurement of the latent trait in standard deviation units (theta), with higher values indicative of higher levels of autistic traits

In addition to calculating internal consistency as a function of the overall scale (i.e. with Cronbach alpha), the precision (reliability) of the scale across the range of possible scores was considered by computing the total information curve (TIC) using IRTPRO software, and then transforming TIC values to reliability using the formula, *Reliability* = 1 − (1/*Information)*. Given the polytomous (ordinal) nature of the response scale, Samejima's graded response model [42] was used for the data. Ideally, the TIC shows relatively high precision (reflected by higher TIC/reliability values) across a wide range of scores as opposed to a narrow range but is often weaker at the end-points which are the measurement boundaries [43]. The plotted TIC (transformed to reliability, see Fig. 2) suggested a respectable level of precision across a broad range scale scores but was weakest for lower levels of autistic traits. Analyses of subscales are reported in Additional file 1: Figure S1.

#### Convergent validity

Convergent validity was assessed by correlating CATI scores with the AQ and BAPQ scores. At the total score level, the CATI correlated highly with both the AQ (*r* = .79, *p* < .001, 95% CI [.77, .81]) and the BAPQ (*r* = .80, *p* < .001, 95% CI [.78, .82]). However, the correlations were not so high as to suggest redundancy. Convergent validity of the CATI subscales is examined in Study 3.

#### Ability for the CATI to discriminate between autistic and non-autistic individuals

##### Recruitment of additional autistic participants

Next, we evaluated the criterion-related validity associated with the CATI and its contemporaries, the AQ and BAPQ. To aid with these analyses, additional autistic participants were recruited into the Study 2 sample. This was achieved by re-opening the study on Prolific Academic to individuals who had identified as autistic in their Prolific Academic profile (several non-autistic participants were inadvertently recruited during this process, increasing the size of this group slightly as well). To maximise recruitment, autistic participants from Study 1 were also eligible for recruitment. Otherwise, the same exclusion criteria applied as in earlier parts of this investigation.

Overall, 90 additional participants were recruited, increasing the total sample size to 1209 participants. The expanded sample now consisted of 56 participants (23 male, 28 female, 1 intersex, 4 sex not given) self-reporting having received a formal diagnosis of ASD, 77 participants (42 male, 35 female) self-identifying as autistic without having received a formal diagnosis, and 1076 participants (535 male, 532 female, 9 sex not given) reporting not having received a formal diagnosis of ASD or otherwise self-identifying as autistic (i.e. non-autistic). Of the 90 additional participants, 40 were returning participants from Study 1 and accounted for 3.31% of the total sample. Participants ages ranged 18–75 years (*M* = 36.87, SD = 12.54). No additionally recruited participant met any exclusion criteria.

##### Descriptive statistics

**Fig. 3:**
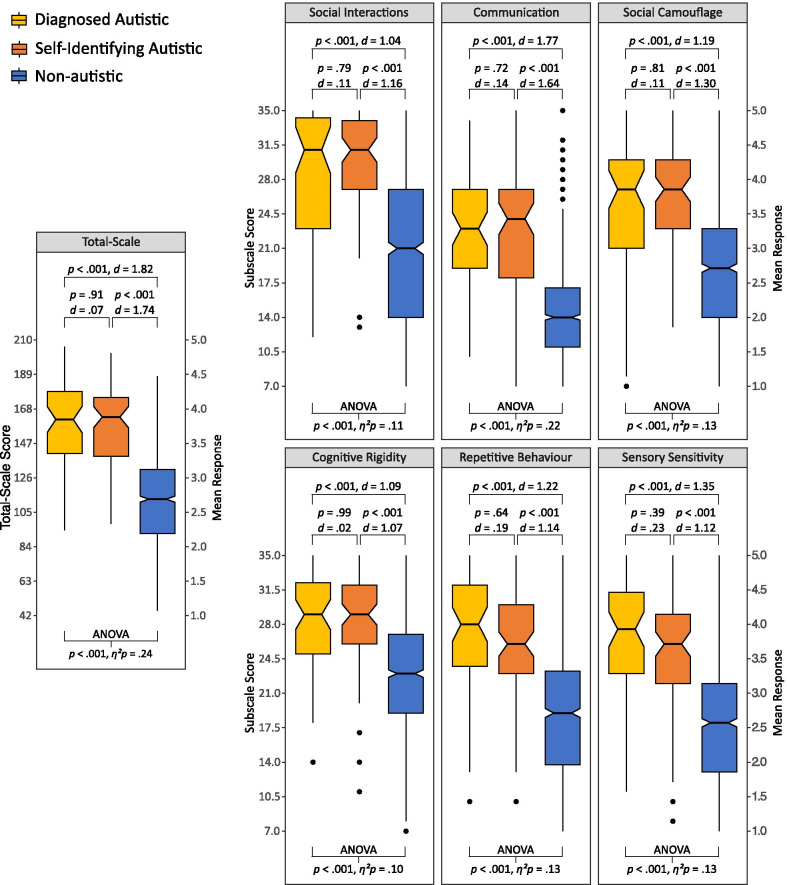
Box-and-whisker summaries of CATI total scores and subscale scores divided by autism group. The mid-line represents the group median, the indents (or ‘notches’) represent the 95% confidence interval of the median, the box represents the inter-quartile range, and the whiskers extend to the furthest score within the inter-quartile range multiplied by 1.5. All observations outside of this range are individually represented by dots. ANOVAs examining group differences are reported at the bottom of each panel and follow-up post hoc Tukey’s *t* tests reported at the top of each panel

A summary of CATI total-scale and subscale scores along with the subsequent analyses is outlined in Fig. 3. Data were first analysed using a repeated measures ANOVA that included participant group as a between-subjects factor (three levels: diagnosed, self-identifying, and non-autistic) and subscale as a within-subjects factor (six levels: one per subscale). The analysis revealed a main effect of participant group, *F*(2, 1206) = 186.66, *p* < .001, *η*^2^_p_ = .24. Post hoc Tukey’s *t* tests indicated that there was no measurable difference in CATI scores between the diagnosed and self-identifying autistic groups (*p* = .91, *d* = .07) but that CATI scores for both groups exceeded the scores observed for the non-autistic group (both *p*s < .001, both *d*s > 1.74). This suggests that there is little difference between individuals who have not been diagnosed, but otherwise identify as autistic, and those who also report having a formal diagnosis, with respect to the dimensions measured by the CATI.^4^

A main effect of subscale, *F*(5, 6030) = 66.00, *p* < .001, *η*^2^_p_ = .05, and interaction between subscale and participant group, *F*(10, 6030) = 3.07, *p* < .01, *η*^2^_p_ < .01, was also found. Effects involving the subscale factor are not entirely surprising given that the subscales were not explicitly designed to produce directly comparable scores and, given that direct comparisons between the factors are not inherently meaningful, ANOVAs and Tukey’s post hoc *t* tests examining participant group differences were conducted separately for each subscale (summarised in Fig. 3). Each ANOVA returned a significant result, and follow-up Tukey’s *t* tests were uniform in suggesting that the two autistic groups were comparable on each subscale, and both showed elevated scores relative to the non-autistic group.

**Fig. 4:**
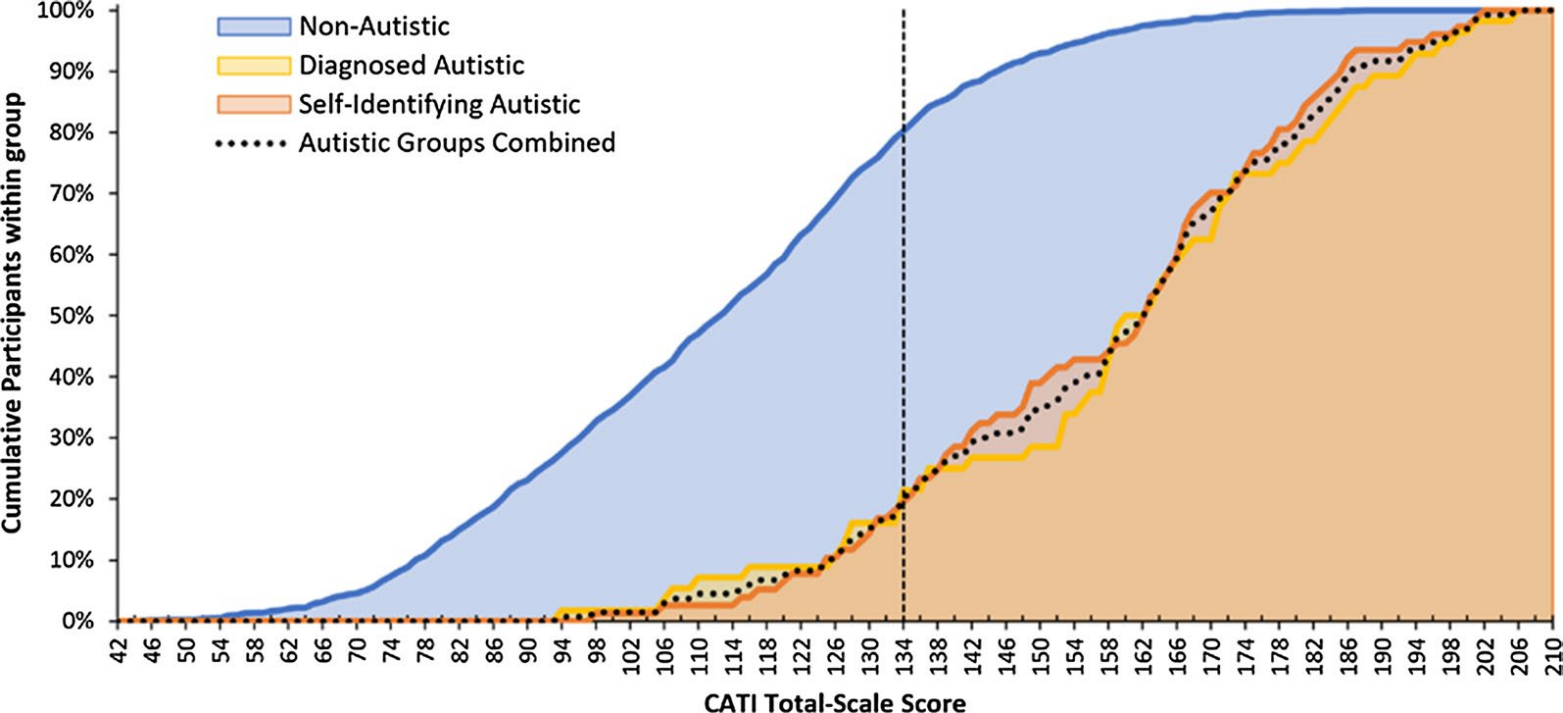
Cumulative distributions of CATI total-scale scores as a function of autism group. Vertical line represents the proposed total-scale score cut-off, 134

Finally, to examine the distribution of total-scale CATI scores in each autism group, we plotted the frequency of total-scale CATI scores for each participant group in Fig. 4. As expected, autistic individuals tended to dominate the higher end of the distribution and were rarer at the lower end.

### Logistic regression

Next, a series of logistic regression analyses was conducted to evaluate further the criterion-related validity of the CATI, in comparison to the AQ and the BAPQ. Given that we did not identify any significant mean differences between the self-identifying and diagnosed autistic participants on the CATI scales, we collapsed together these two groups into a single ‘autistic’ category (*n* = 133) for the purposes of conducting the binary logistic regression (0 = non autistic; 1 = autistic). All six subscales were entered as predictors into the model in a single step (method: enter).

First, three analyses were conducted where the subscales of CATI, AQ and BAPQ were entered as predictors into separate models to determine the ability to predict autistic status for each questionnaire independently. The overall summary of each model as well as the coefficients associated with each predictor in each model are outlined in Table 5. Critically, all six subscales in the CATI yielded significant independent contributions to the logistic regression equation. In contrast, two of the five AQ subscales did not yield significant independent contributions, with *Imagination* notably showing *p* = .655, and nor did one of the three BAPQ subscales, *Aloof Personality,* showing *p* = .396. Furthermore, the CATI predicted the greatest amount of variance, with Nagelkerke’s pseudo *R*^2^ indicating that it accounted for 48.2% of the total variance in autistic status. Specifically, the CATI accounted for 3.5% more variance than the AQ (or a relative percentage increase of 8%), and 10.4% more variance than the BAPQ (or a relative percentage increase of 28%). Additional hierarchical logistic regressions (see Additional file 1: Table S3) revealed that this additional variance predicted, though small in the case of the AQ, was statistically significant.

**Table 5:** Model summaries of several logistic regression analyses predicting autistic status using the CATI, AQ and BAPQ, and standardised coefficients for each model’s predictors

	*β*	Wald test		Odds ratio	95% CI for odds ratio	
		Wald	*p*		Lower	Upper
CATI: *df* = 6, *χ*^2^ = 332.95, *p* < .001, Nagelkerke’s pseudo *R*^2^ = .482						
Constant	− 3.446	183.20	< .001	.000	.000	.000
Social interactions	.390	5.70	.017	1.049	1.009	1.090
Sensory sensitivity	.371	5.90	.015	1.056	1.011	1.103
Repetitive behaviour	.435	6.83	.009	1.065	1.016	1.116
Communication	.915	46.92	< .001	1.179	1.125	1.236
Cognitive rigidity	.340	4.09	.043	1.062	1.002	1.126
Social camouflage	.339	4.23	.040	1.053	1.002	1.106
AQ: *df* = 5, *χ*^2^ = 305.84, *p* < .001, Nagelkerke’s pseudo *R*^2^ = .447						
Constant	− 3.223	166.03	< .001	.000	.000	.000
Social skill	.305	2.97	.085	1.049	.993	1.108
Attention switching	.666	16.28	< .001	1.142	1.071	1.218
Attention to detail	.290	6.47	.011	1.059	1.013	1.107
Communication	1.054	32.01	< .001	1.216	1.136	1.301
Imagination	.053	.20	.655	1.011	.963	1.062
BAPQ: *df* = 3, *χ*^2^ = 253.46, *p* < .001, Nagelkerke’s pseudo *R*^2^ = .378						
Constant	− 2.990	212.35	< .001	.000	.000	.000
Aloof personality	.127	.72	.396	1.010	.987	1.035
Pragmatic language	1.038	57.02	< .001	1.116	1.085	1.148
Rigidity	.771	28.65	< .001	1.079	1.049	1.109

### Identifying a relevant threshold

While the CATI was primarily developed to assess autistic traits in non-autistic individuals and not to be used as a diagnostic tool, for some research purposes, a total-score cut-off that best discriminates between autistic and non-autistic individuals may be desirable. We identified a CATI total-scale score of 134 and above as the ideal cut-off for classifying individuals as autistic, as this value maximised the sum total of sensitivity (true positive rate; 82.71%) and specificity (true negative rate; 79.00%). A total score of 134 corresponds to a mean item response of 2.79 on the CATI five-point Likert scale.

To provide a valid comparison of the relative accuracy of this cut-off with the AQ and BAPQ, we used an identical method to identify cut-offs for these scales using our data (rather than directly compare to cut-offs and observations made using different samples). We next calculated Youden’s Index for each scale to provide an overall measure of predictive ability that combines sensitivity and specificity into a single index that ranges from 0 to 1 (greater values indicate greater predictive ability). Using the previously identified cut-off of 134, a Youden’s Index of .62 was observed for the CATI. For the AQ and BAPQ, we identified that the sum of sensitivity and specificity was maximised using cut-offs of 132 and 134 respectively, which translated to Youden’s Index values of .59 and .56, respectively. Thus, consistent with the results of the logistic regression analyses, these values also suggest that the CATI has greater predictive ability than previous measures. A full table of sensitivity and specificity values, Youden’s indices, and cut-offs for the total-scale and each subscale of the CATI, AQ and BAPQ can be found in Additional file 1: Table S4.

### Examination of sex differences

Our final analyses explored the possibility of sex differences on the CATI scales. Autism is more common in males than females with a ratio of 3:1 [44] and males also tend to show elevated levels of autistic traits on measures like the AQ [4]. However, there is a growing awareness that uniquely female presentations of autism may exist and actual sex differences in prevalence may be lower than reported [45, 46]. For example, females are more likely than males to be aware of their differences and engage in ‘masking’ behaviours and other strategies to fit in with their peers [33, 34], which may be potentially enabled by females’ greater communicative abilities [31, 47, 48].

Consequently, it is prudent to determine whether sex differences are also revealed using the CATI in a sample that is relatively broadly representative, examining differences both in terms of factor structure, and total-scale and subscale mean scores. To this end, analyses were conducted with the sample described for this study before additional autistic participants were added to the sample (*N* = 1109), excluding individuals who did not explicitly report their sex as male or female. A factorial invariance analysis was initially conducted to determine if the CATI factor structure varied as a function of sex. As can be seen in Table 6, the finding of scalar (strong) invariance shows that the CATI six-factor two-bifactor model fitted the data for the male and female participants comparably. Additionally, the CATI also met the criteria for strict invariance. This means male and female participants can be directly compared and the comparisons meaningfully interpreted.

**Table 6:** Results of a multi-group factorial analysis assessing measurement and structural invariance of the CATI as a function of participant sex

Model	*χ*^2^	*df*	CFI	RMSEA (90% CI)	Δχ^2^	Δ*df*	ΔCFI	ΔRMSEA	Decision
Configural Invariance	3937	1552	.989	.053 (.051–.055)	–	–	–	–	Accept
Metric (weak) invariance	4293	1712	.988	.052 (.050–.054)	356	160	.001	− .001	Accept
Scalar (strong) invariance	5114	1754	.985	.059 (.057–.061)	821	42	.003	.007	Accept
Residual (strict) invariance	5464	1796	.983	.061 (.059–.063)	350	42	.002	.002	Accept

ΔCFI > .01 and ΔRMSEA > .015 are indicative of a violation of the invariance assumption [49, 50]

*CFI* comparative fit index, *RMSEA* root mean square error of approximation

**Fig. 5:**
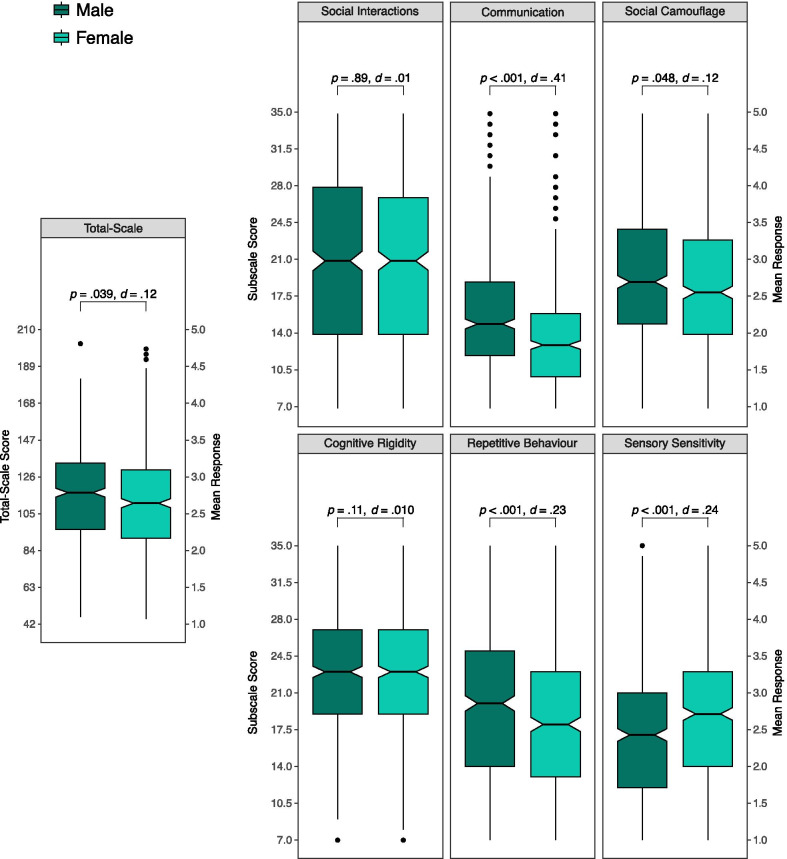
Box-and-whisker summaries of CATI total scores and subscale scores divided by sex. The mid-line represents the group median, the indents (or ‘notches’) represent the 95% confidence interval of the median, the box represents the inter-quartile range, and the whiskers extend to the furthest score within the inter-quartile range multiplied by 1.5. All observations outside of this range are individually represented by dots. Comparisons are student *t* tests

Next, we compared mean group differences in total-scale and subscale scores as a function of sex (illustrated in Fig. 5). First, a repeated measures ANOVA with sex (two levels: male and female) as a between-subjects factor and subscale (six levels) as a within-subjects factor was performed. The analysis revealed main effects for both subscales, *F*(5, 5535) = 344.75, *p* < .001, *η*^2^_p_ = .24 (which follows, given the same finding for this factor in the previous analysis comparing autism groups), and sex, *F*(1, 1107) = 4.28, *p* = .039, *η*^2^_p_ < .01, with the latter analysis reflected a small elevation in CATI scores in males relative to female. Of greater interest was a significant interaction between sex and subscale, *F*(5, 5535) = 17.86, *p* < .001, *η*^2^_p_ = .02. The interaction was followed up with a series of t-tests examining sex differences for each subscale, with the results of each comparison (including total-scale scores) reported in Fig. 5. Males showed significantly higher scores on the *Communication* (*d* = .41) and *Repetitive Behaviour* (*d* = .23) subscales. By contrast, *Sensory Sensitivity* was greater in women compared to men (*d* = .24). A small elevation in *Social Camouflage* scores was noted for females relative to males (*d* = .12), but the effect was not significant when taking into consideration corrections for multiple comparisons. Taken together, these comparisons suggest that sex differences in autistic traits are not uniform across the different trait dimensions measured by the CATI, a finding that is in line with the notion that autism presents differently in males and females [45, 46].

#### Discussion

In Study 2, we verified the six-factor 42-item version of the CATI identified in Study 1 in a new sample of participants. Fit indices supported the six subscales structured within a bifactor model with second-order factors separately encompassing items loading on the social and non-social subscales. Internal consistencies calculated for the total scale and six subscales all indicated that this model was well-supported from a psychometric standpoint. Internal consistency across the scale was also acceptable, with lower measurement precision only observed at the ends of the continuum of scale scores as is typical for such measures. Furthermore, when examining group means for autistic and non-autistic participants separately, we found that each subscale, in addition to the total-scale, could consistently distinguish autistic and non-autistic participants. Interestingly, significant differences were not found between the group means of diagnosed and self-identifying, undiagnosed, autistic participants on any of the measures used in Study 2. This suggests that those participants who self-identify as autistic are, for practical purposes, no different to those who have received a diagnosis. Of course, it cannot be ruled out that relatively nuanced differences exist between the groups that are simply not detectable by any of the measures used in the study.

Using logistic regression, Study 2 also revealed that the CATI has slightly more predictive power than the AQ and BAPQ for classifying autistic (diagnosed and self-identifying) and non-autistic participants. A notable strength of the new measure if that each of the subscales demonstrates predictive utility, which cannot be said about the subscales of the AQ and BAPQ. While the CATI’s predictive performance is only slightly higher numerically in terms of Nagelkerke’s *R*^2^, even incremental improvements are valuable, especially when observing particularly large sample sizes.

Finally, multi-group factorial analysis showed that the factor structure of the CATI was invariant between sexes, indicating that subscale scores for males and females can be directly compared. In examining total-scale means, males were found to have slightly higher CATI scores than females, an outcome in line with similar findings for the AQ [2] and BAPQ [39]. At the subscale level, several statistical differences were identified. Males showed higher levels on the *Communication* and *Repetitive Behaviours* subscales which are therefore the likely drivers of the total-scale sex difference. Conversely, *Sensory Sensitivity* was found to be higher for female participants, and follows reports of higher scores on the Sensory Perception Quotient for females relative to males [51]. To our knowledge, this is the first time that a broad measure of autistic traits has shown variations in sex differences across multiple subscales, and further establishes the CATI as a psychometric measure that is relatively sensitive to the subtler differences in autistic trait presentation between males and females compared to existing scales.

## Study 3: Convergent validity for CATI subscales

In Study 2, the total-scale scores obtained from the AQ and BAPQ—scales that also assess a range of autistic trait dimensions—correlated highly with the total-scale score from the CATI, thereby establishing its convergent validity. For Study 3, we aimed to establish convergent validity for the CATI subscales as well by administering the CATI alongside six sets of items taken from other established scales, each of which corresponded conceptually to a subscale from the CATI. Specifically, the (sub)scales selected for each CATI subscale were:

- For *Social Interactions*, the Social Skill subscale of the AQ (as defined by Russell-Smith et al.) [23]
- For *Communication*, the Communication subscale of the AQ (as defined by Russell-Smith et al.) [23]
- For *Repetitive Behaviours*, the Repetitive Behaviours subscale of the Adult Repetitive Behaviours Questionnaire [20]
- For *Cognitive Rigidity*, the Insistence on Sameness subscale of the Adult Repetitive Behaviours Questionnaire
- For *Sensory Sensitivity*, the Hyper-sensitivity items from the Glasgow Sensory Questionnaire [21]
- For *Social Camouflage*, the total score from the Camouflaging Autistic Traits Questionnaire [52]

### Methods

#### Participants

Two hundred and ten individuals who had not previously taken part in Study 1 or 2 were recruited into Study 3. As with the previous studies, prior to any analysis, participants were removed from the dataset if one or more of the following conditions were observed: (a) self-reported a primary language other than English (*n* = 0); (b) failed any of the attention checks built-in to the questionnaire (*n* = 8); or (c) completed the questionnaires too quickly (i.e.  < 5 min; *n* = 1). For the remaining 202 participants, there was a relatively even male-to-female ratio of participants (103 male, 99 female) similar to the previous studies. Participants ages ranged from 18 to 71 years (*M* = 33.71, SD = 11.81). Within the sample, 3 participants (1 male, 2 female) reported having previously received a formal diagnosis of ASD. The proportion of diagnosed autistic participants in the sample (1.49%) is similar to current USA population estimates [1 in 59, or 1.69% (30)]. A further 4 participants (2 male, 2 female) reported that they self-identified as autistic but had not received a formal diagnosis. The remaining 195 participants (100 male, 95 female) did not report having received a diagnosis of autism or otherwise self-identifying as autistic (i.e. non-autistic).

### Materials

#### CATI

The version of the CATI described in Study 2 was also used in this study.

#### AQ (social and communication)

A description of the AQ can be found in the Study 2 materials. For this present study, items from the AQ that corresponded to the *Social Skill* and *Communication* factors identified in Russell–Smith [23] were administered to participants (in the order they appeared in the original AQ), and all other items were excluded.

#### Glasgow Sensory Questionnaire

The Glasgow Sensory Questionnaire (GSQ) [21] is a 42-item self-report questionnaire that assesses seven different sensory domains (visual, auditory, gustatory, olfactory, tactile, vestibular and proprioceptive) with an equal number of items for each sensory domain, and evenly divided between items that assess hyper-sensitivity and hypo-sensitivity. Items take the form of questions (“*Do bright lights ever hurt your eyes/cause a headache?*”) and a five-point Likert scale (‘Never’, ‘Rarely’, ‘Sometimes’, ‘Often’, ‘Always’) is used for responses. For the present study, we retained the 21 items that assess sensory hyper-sensitivity as the CATI’s *Sensory Sensitivity* subscale was also composed of hyper-sensitivity items. These items were presented in their original order and the remaining 21 hypo-sensitivity items were not administered. Each item was scored 0–5, with higher scores reflecting more pronounced sensitivity.

#### Adult Repetitive Behaviours Questionnaire

The Adult Repetitive Behaviours Questionnaire-2 (ARBQ-2A) is a 20-item self-report questionnaire that assesses repetitive and restricted behaviours and interests [20]. Items take the form of questions (e.g. “*Do you like to arrange items in rows or patterns?*”) and responses are made using a 3 or 4-point Likert scale, with response options varying according to the question, and higher scores corresponding to greater levels of autistic traits.^5^ For the present study, we administered the six items associated with the *Repetitive Motor Behaviours* subscale and eight items associated with *Insistence on Sameness* subscale [20] and presented the items of these subscales in their original order. Items that did not correspond to either of these two subscales were not administered.

#### Camouflaging Autistic Traits Questionnaire (CAT-Q)

The Camouflaging Autistic Traits Questionnaire (CAT-Q) is a 25-item self-report questionnaire that assesses strategies and behaviours to compensate or mask autistic characteristics in a social context [52]. Items take the format of statements (e.g. “*I always think about the impression I make on other people*”) with responses made on a seven-point Likert scale that ranges from “Strongly Disagree” to “Strongly Agree”. Higher scores indicate greater levels of camouflaging traits, with five items negatively keyed.

#### Procedure

The procedure was largely identical to that used for Studies 1 and 2. The main difference was that, instead of the full AQ and BAPQ being administered alongside the CATI, the partial versions of the AQ, ARBQ-2 and GSQ, and full CAT-Q were administered instead. Questionnaire order was randomised for each participant, while item order adhered to the order documented for each questionnaire. Finally, one attention-check item, similar to those used in Study 1 and 2, was placed at the mid-point of each of the five questionnaires with the item phrased to fit each questionnaire’s statement and response format. All other aspects of the procedure were identical to that used for Study 1 and 2.

### Results and discussion

Pearson correlations were used to compare each CATI subscale with its conceptual counterpart, as outlined earlier. The correlations are outlined in Table 7, with the key comparisons (e.g. CATI *Social Interactions* and AQ *Social Difficulty*) highlighted in shaded cells. The alpha for the significance level *p* = .05 following a Bonferroni adjustment for multiple [36] comparisons is *p* = .00139, which was met in every key comparison and the majority of the others. Critically, each CATI subscale score was most strongly correlated with the expected comparison score, establishing convergent validity for the subscales in addition to the total-scale scores described in Study 2.

**Table 7:** Pearson correlations between the CATI subscale scores and corresponding scores obtained from the AQ, ARBQ-2, GSQ, and CAT-Q, and internal consistencies (Cronbach alpha) for each (sub)scale

	CATI					
	Social interactions (*α* = .95)	Communication (*α* = .83)	Repetitive behaviour (*α* = .86)	Cognitive rigidity (*α* = .85)	Sensory sensitivity (*α* = .83)	Social camouflage (*α* = .87)
AQ—Social difficulty (*α* = .93)						
*r*	*.916*	.449	.284	.275	.446	.406
*p*	< *.0001*	< .0001	< .0001	< .0001	< .0001	< .0001
AQ—Communication/mindreading (*α* = .77)						
*r*	.486	*.751*	.337	.218	.383	.417
*p*	< .0001	< *.0001*	< .0001	.0018	< .0001	< .0001
ARBQ-2A—Repetitive motor behaviour (*α* = .77)						
*r*	.210	.247	*.701*	.381	.470	.466
*p*	.0027	.0004	< *.0001*	< .0001	< .0001	< .0001
ARBQ-2A—Insistence on sameness (*α* = .76)						
*r*	.306	.346	.515	*.679*	.583	.406
*p*	< .0001	< .0001	< .0001	< *.0001*	< .0001	< .0001
GSQ—Hyper-sensory sensitivity (*α* = .89)						
*r*	.387	.361	.439	.412	*.773*	.419
* p*	< .0001	< .0001	< .0001	< .0001	< *.0001*	< .0001
CATQ—Total scale (*α* = .92)						
* r*	.614	.459	.595	.379	.528	*.805*
*p*	< .0001	< .0001	< .0001	< .0001	< .0001	< *.0001*

Italicised values indicate the key comparisons between a subscale from the CATI and a similar (sub)scale from an established measure

Internal consistency (Cronbach alpha) was also each for subscale included in the correlation analysis and is also reported in Table 7. While all (sub)scales showed acceptable levels of internal consistency, the CATI subscales tended to outperform the comparison scales with the exception of *Sensory Sensitivity* and *Social Camouflage*, which were slightly lower in Cronbach alpha than their comparisons. However, considering that the internal consistency was still considerable high (.83 vs .89 and .87 vs .92, respectively) and is achieved with far fewer items than for their contemporaries (14 and 18 items fewer respectively), it could be argued that the small drop in internal consistency is a reasonable trade-off for increased efficiency.

### General discussion

#### Summary

This paper describes the development and psychometric evaluation of a new scale for measuring autistic traits, the Comprehensive Autistic Trait Inventory (CATI). Following initial item creation, exploratory factor analyses conducted on a large sample of adults identified a six-factor structure with factors spanning *Social Interactions*, *Communication*, *Sensory Sensitivity*, *Repetitive Behaviour*, *Social Camouflage*, and *Cognitive Rigidity*. Seven items were then selected from each factor to create the 42-item CATI. Confirmatory factor analyses of the six-factor model suggested several models with adequate psychometric properties but that the best model for the data was one that incorporated two additional second-order bifactors, with one covering the items that loaded on the social factors (*Social Interactions*, *Communication*, *and Social* Camouflage) and one covering the items that loaded on the non-social factors (*Repetitive Behaviour*,* Cognitive Rigidity*,* and Sensory* Sensitivity). This model seems apt, given the diagnostic criteria for autism also largely separates into social and non-social dimensions [19], and provides researchers with the option of calculating both the relatively domain-specific subscale scores as well as scores for the two bifactors, which provide broad measures of social and non-social autistic traits.

Furthermore, internal consistency of the total-scale and each of the subscales exceeded the threshold required for practical use and interpretation. Binomial logistic regression analyses found that the scale improved incrementally on the AQ and BAPQ in classifying autistic and non-autistic individuals. Additionally, multi-group factorial analyses comparing male and female participants found evidence for measurement invariance, indicating that scores can be compared between males and females. Finally, excellent convergent validity was demonstrated for each of the CATI subscales, with each correlating strongly with a conceptually similar measure derived from another questionnaire.

#### Strengths

Overall, the new scale is a compelling alternative to other measures of autistic traits, such as the AQ [2], the BAPQ [12], the SRS-2 [11]. The CATI provides separate measures of six different trait dimensions associated with autism, one more than the proposed subscales of the AQ and SRS-2, and three more than the BAPQ. This means that users of the CATI will have access to a larger range of trait dimensions than was previously possible with the administration of a single scale. In addition, as a total-scale score obtained using the CATI encompasses a wider variety of autistic trait dimensions than previous measures, it is potentially more accurate at differentiating overall ‘low’ and ‘high’ trait individuals and should be less prone to measurement blindspots. For example, an individual particularly high in traits relating to sensory sensitivity and repetitive behaviours might only show moderate trait levels on the BAPQ as this measure does not capture those trait dimensions well.

Furthermore, the CATI assesses a breadth of different trait dimensions without becoming unnecessarily ‘bloated’, coming in at 42 items in length, comparing favourably to the AQ (50 items) and the SRS-2 (65 items), and containing only six items more than the BAPQ (36 items) while capturing three additional trait dimensions. Importantly, this efficiency does not compromise internal reliability, as we found the internal reliability of each CATI subscale to exceed .80, comparable to the BAPQ (which has 12 items per subscale) and superior to the original AQ subscales, which, in Study 2, provided Cronbach alpha values between .69 and .74 for four of the five subscales.

While it is important to reiterate that the development of the CATI was focussed on its ability to measure traits associated with autism in non-autistic individuals, it is nonetheless notable that the CATI also appears to be better at distinguishing autistic and non-autistic individuals compared to other measures of autistic traits. Binomial logistic regression attempting to categorise our Study 2 participants into non-autistic and autistic (either diagnosed or self-identifying) groups revealed that all CATI subscales were significant contributors to the predictive model. The same could not be said for the AQ and BAPQ as it was clear that there were subscales that appeared to contribute little (namely, *Imagination* in the AQ, and *Aloof Personality* in the BAPQ). More importantly, the overall logistic binomial regression models indicated that the CATI explained more variance (.482) than either the AQ (.447) or BAPQ (.378). Between the AQ and CATI, this works to be a difference of .035 in variance explained, or a non-trivial 7.83% increase moving from the AQ to the CATI. Supplementing the regression analyses were examinations of sensitivity and specificity. With respect to sensitivity and specificity, Youden’s Index, which combines these into a single index, demonstrated superior autistic/non-autistic prediction for the CATI (.62) compared to the AQ (.59) and BAPQ (.56).

#### Limitations and future directions

Despite the considerable strengths of our new measure, there remain aspects of the CATI that require further attention to verify the extent of its usefulness for researchers. Perhaps most notable is our reliance on self-report for participant’s autism ‘status’. While self-report facilitates the ability to recruit participants into the study, it is possible that a number of self-reported non-autistic participants are in fact autistic and, similarly, some self-reported autistic participants may not actually meet the criteria for clinically defined autism. We partially addressed this issue by allowing autistic participants to specify if they had been formally diagnosed or had not been diagnosed but otherwise self-identified as autistic. If self-identifying autistic participants were systematically inaccurate in certain aspects of their self-diagnosis we would have expected to see disparities between the two autism groups but, instead, we found a remarkable level of similarity between the two groups across all six CATI subscales. Regardless, there is a need for additional discrimination analyses to be conducted using a large sample of clinically confirmed autistic and non-autistic individuals to confirm the reported discriminability of the CATI.

On a similar note, the CATI has yet to be tested for its ability to discriminate autism from other clinical conditions. This is particularly important given the known high comorbidity of autism with other conditions, and a crucial next-step in further evaluating the CATI will be establishing its usefulness in identifying autistic traits specifically (as opposed to traits associated with other conditions). Furthermore, the current paper does not establish measurement invariance for the CATI with respect to autism status, meaning that it is uncertain whether the same factor structure applies to both autistic and non-autistic individuals, as well as to groups of individuals with other conditions. Establishing measurement invariance requires a much larger sample of autistic participants than was available at the time of testing and, ideally, would include participants whose autism diagnosis (and other diagnoses) could be clinically confirmed.

Furthermore, with regard to the predictive capacity to identify autism explored by the regression analyses in Study 2, it should be strongly re-iterated that these outcomes are preliminary. Given the relatively small sample of autistic participants in the study and that diagnoses were not confirmed, the suggested cut-offs should be used cautiously until they can be validated with a larger, clinically confirmed sample of autistic individuals. Of course, we recognise that even preliminary cut-offs may be of interest to some researchers, hence their inclusion in the current paper. Relatedly, the odds ratios reported in the logistic regression, while significant, are also relatively small. While the odds ratios are somewhat better for the CATI compared to the AQ and BAPQ, the CATI may still have difficulty distinguishing between a non-autistic individual with high levels of autistic traits and a clinically diagnosed autistic individual. Consequently, we remind readers that the scale is not intended to be a diagnostic tool but to provide useful measures of trait dimensions across the general population.

Though the CATI captures a broad range of trait dimensions, some readers may be surprised at the absence of specific subscales that relate to “insistence on sameness” and/or “restricted interests”. As outlined in Study 1 and the Additional file 1: Table S2, we developed a number of items on these themes in the expectation that they would form a factor which we referred to as ‘monotropic mindset’ in the exploratory factor analysis. However, we found that these items tended to load on the cognitive rigidity and repetitive behaviours factors instead of forming an independent factor of their own. We did experiment with forcing additional factors in the exploratory analyses and did eventually find the expected factor but there was no psychometric support for such models. One reason for this could be that the particular items that we tested were too dissimilar and did not readily form a cohesive construct. Alternatively, the absence of such a factor may potentially indicate that this dimension is relatively ‘weak’ compared to the other, more pronounced, dimensions in the general population. Either way, this is a potential area of focus for a revised version of the CATI in the future as additional item development and refinement may produce a subscale with psychometric properties similar to the existing subscales.

Somewhat relatedly, some readers may question the inclusion of the *social camouflage* subscale given that camouflaging is not currently a diagnostic criterion for autism. Indeed, attempting to diagnose autism where camouflaging is a prerequisite could potentially exclude individuals who cannot or choose not to camouflage. While justifications were made for the inclusion of camouflaging traits while describing the pilot version of the CATI, we understand that there may be circumstances where researchers might prefer to leave out this subscale when using the CATI in certain clinical contexts.

Regarding sensory sensitivity, the CATI includes items relating to hyper-responsiveness to sensory stimuli but not hypo-responsiveness, which is also a relatively common characteristic of autism [53]. The focus on hyper-responsiveness stems from related work that has failed to find reliable unique contributions of hypo-responsiveness using self-report questionnaires. For example, principal components analysis of the Glasgow Sensory Questionnaire using responses from participants recruited from the general population revealed only a single component on which all items loaded despite the questionnaire consisting of equal numbers of items relating to hyper- and hypo-sensitivity to sensory stimuli [21]. This pattern was supported by data from the Dutch translation of the scale, where a moderate positive correlation was reported between the total scores of the hyper- and hypo-sensitivity items [54]. Furthermore, significantly higher levels of hyper-responsiveness in autistic compared to non-autistic women have been reported using a revised version of the Sensory Perception Quotient, but no differences in hypo-sensitivity were reported [51]. Consequently, in the interest of brevity the development of the current version of the CATI was restricted to items relating to hyper-responsiveness to sensory stimuli. Similar to the issue of items assessing ‘monotropic mindset’, the development of hypo-sensitivity items would be an area worthy of future investigation.

Finally, while the CATI is comprehensive in the breadth of trait dimensions assessed, we acknowledge that other measures may provide a more fine-grained examination of specific dimensions. For example, the Glasgow Sensory Questionnaire allows for greater distinction between the individual sensory domains than is possible with the CATI. Similarly, the Multidimensional Social Competence Scale [55] measures specific social competencies (i.e. *social inferencing*, *empathic concern*, etc.) and the Camouflaging Autistic Traits Questionnaire [52] breaks down camouflaging behaviours into several distinct subtypes (i.e. *compensation*, *masking*, and *assimilation*). Consequently, if the influence of a specific trait dimension is of interest, specialised measures such as these may be preferable to broader measures. However, when capturing a range of trait dimensions is desirable, the CATI is well-suited for the task, additionally providing a total-scale that is more broadly representative of autism than existing general autistic trait measures.

### Conclusion

Autistic traits will likely be assessed to conduct autism-related research and help improve our understanding of autism more broadly for many years to come. Therefore, it is essential that researchers seeking to measure and examine the influence of autistic traits have the most appropriate tools to accomplish their aims. There is no denying that existing measures have been critical to conducting autism research over the previous 2 decades, but furthering our continued understanding of autism depends on modern scales that have been developed using established psychometric techniques combined with our current understanding of autism spectrum conditions. The CATI picks up where previous measures, like the AQ and BAPQ, left off and builds upon their existing strengths (e.g. quantifying social difficulties) while simultaneously covering blindspots that have emerged in the intervening years since their development (e.g. sensory sensitivity). Though the CATI does not cover every aspect of autism, it is currently the most comprehensive self-report measure developed to date. We hope that researchers with an interest in measuring autistic traits consider the utility of this new measure and that it proves as useful as the scales and questionnaires that preceded it.

## Supplementary Information

**Additional file 1.** Supplementary tables and figures.

**Additional file 2.** PDF versions of the CATI and scoring key.

## Data Availability

The datasets generated and analysed during the current study are not publicly available but may be obtained from the corresponding author on reasonable request.

## Footnotes

1 Internal consistencies are also retrospectively reported for the social and non-social bifactors identified using confirmatory factor analysis in Study 2.

2 Where discretion was used to ignore certain items and select viable alternatives, the reasoning is outlined alongside the initial pattern matrix in Additional file 1: Table S2.

3 Readers may notice that the number of exclusions is considerably lower than what was reported for Study 1. We suspect that a certain portion of participants recruited through web-platforms such as Prolific Academic aim to sign up early to new studies and complete them as quickly as possible to earn monetary rewards. As Study 2 did not allow participants from Study 1 to participate, we hypothesise that most of the early, rapid responders were prevented from attempting the second study. We advise researchers to use attention checks and keep a record of the IDs of participants who fail these checks to inform their future studies on online platforms.

4 Similar patterns of group differences were found when t tests were conducted on the total-scale scores for the AQ, *p* = .89, *d* = .03, and the BAPQ, *p* = .29, *d* = .19.

5 For non-clinical samples, the scale’s authors recommend recoding any ‘4’ responses as a ‘3’.
