# Correction to: The Comprehensive Autistic Trait Inventory (CATI): development and validation of a new measure of autistic traits in the general population

**DOI:** 10.1186/s13229-021-00475-1

**Published:** 2021-11-08

**Authors:** Michael C. W. English, Gilles E. Gignac, Troy A. W. Visser, Andrew J. O. Whitehouse, James T. Enns, Murray T. Maybery

**Affiliations:** 1 School of Psychological Science, University of Western Australia, Perth, WA Australia; 2 Telethon Kids Institute, Perth, WA Australia; 3 Department of Psychology, University of British Columbia, Vancouver, BC Canada

## Correction to Molecular Autism (2021) 12, 37 https://doi.org/10.1186/s13229-021-00445-7

Following publication of the original article [1], the authors noticed the following two errors:

1. In **Table 1 of the main manuscript**, and **Table S2 of the supplementary material**, the authors described the results of exploratory factor analyses. In both tables, the CATI item labelled “*I would describe myself as a very sensory-sensitive person*” is mislabelled and should read *“I am over-sensitive to bright lighting*”.
  The correct text for this item was used in the supplementary material relating to the distributable version of the CATI, and in the CATI materials on the CATI website.
2. In **the abstract**, the sample sizes of studies 1, 2 and 3 were incorrect and referred to the number of non-autistic participants recruited in each study instead of the overall sample size. These were originally listed as 1119, 1068, and 195, respectively. The correct sample sizes are Study 1 = 1166, Study 2 = 1119, and Study 3 = 202.
  These sample sizes were correct in the main article body and in the additional file of the article.

These errors do not impact the conclusions of the article. The original article and Additional File 1 have been updated. We thank the reader for bringing this to our attention.

## Supplementary Information

**Additional file 1.** Supplementary tables and figures.

Supplementary tables and figures

## References

[1] English MCW, Gignac GE, Visser TAW (2021). The Comprehensive Autistic Trait Inventory (CATI): development and validation of a new measure of autistic traits in the general population. Molecular Autism.

