# Supplementary material for: The Comprehensive Autistic Trait Inventory (CATI): development and validation of a new measure of autistic traits in the general population
Source: Mol Autism. 2021 May 17;12:37. doi: 10.1186/s13229-021-00445-7 (PMC8130295; doi:10.1186/s13229-021-00445-7)
Supplement: Supplementary file 2 — Additional file 2. PDF versions of the CATI and scoring key. [file 13229_2021_445_MOESM2_ESM.zip › CATI - fillable form and automatic scoring.pdf]

CATI

Date: Name/ID:

INSTRUCTIONS

Below is a list of statements relating to various personality traits, behaviours, and characteristics. Using the five response options select the option that best describes you. For items of a social nature, think about situations that do not involve very close friends or family members. Try not to spend too much time thinking about each choice.

REMOVE ALL DATA FROM FORM

Responses saved to this PDF will be visible to others if you share it. If you do not want to share your responses, you can clear everything in the document by clicking this button.

		Definitely Disagree	Somewhat Disagree	Neither Agree nor Disagree	Somewhat Agree	Definitely Agree
1	I often find myself fiddling or playing repetitively with objects (e.g. clicking pens)	○	○	○	○	○
2	I like to stick to certain routines for every-day tasks	○	○	○	○	○
3	I expend a lot of mental energy trying to fit in with others	○	○	○	○	○
4	I am over-sensitive to bright lighting	○	○	○	○	○
5	There are certain activities that I always choose to do the same way, every time	○	○	○	○	○
6	Sometimes I watch people interacting and try to copy them when I need to socialise	○	○	○	○	○
7	I often rock when sitting in a chair	○	○	○	○	○
8	I generally enjoy social events	○	○	○	○	○
9	I look for strategies and ways to appear more sociable	○	○	○	○	○
10	In social situations, I try to avoid interactions with other people	○	○	○	○	○
11	There are times when I feel that my senses are overloaded	○	○	○	○	○
12	There are certain objects that I fiddle or play with that can help me calm down or collect my thoughts	○	○	○	○	○
13	Reading non-verbal cues (e.g. facial expressions, body language) is difficult for me	○	○	○	○	○
14	I like my belongings to be sorted in certain ways and will spend time making sure they are that way	○	○	○	○	○
15	Social interaction is easy for me	○	○	○	○	○
16	When interacting with other people, I spend a lot of effort monitoring how I am coming across	○	○	○	○	○
17	I find social interactions stressful	○	○	○	○	○
18	I am over-sensitive to touch	○	○	○	○	○
19	I can tell how people feel from their facial expressions	○	○	○	○	○
		Definitely Disagree	Somewhat Disagree	Neither Agree nor Disagree	Somewhat Agree	Definitely Agree

		Definitely Disagree	Somewhat Disagree	Neither Agree nor Disagree	Somewhat Agree	Definitely Agree
20	I have a tendency to pace or move around in a repetitive path	◯	◯	◯	◯	◯
21	I feel discomfort when prevented from completing a particular routine	◯	◯	◯	◯	◯
22	I rely on a set of scripts when I talk with people	◯	◯	◯	◯	◯
23	I find it easy to sense what someone else is feeling	◯	◯	◯	◯	◯
24	I am over-sensitive to particular tastes (e.g. salty, sour, spicy, or sweet)	◯	◯	◯	◯	◯
25	I engage in certain repetitive actions when I feel stressed	◯	◯	◯	◯	◯
26	I rarely use non-verbal cues in my interactions with others	◯	◯	◯	◯	◯
27	I often insist on doing things in a certain way, or re-doing things until they are ‘just right’	◯	◯	◯	◯	◯
28	I feel confident or capable when meeting new people	◯	◯	◯	◯	◯
29	Before engaging in a social situation, I will create a script to follow where possible	◯	◯	◯	◯	◯
30	Social occasions are often challenging for me	◯	◯	◯	◯	◯
31	Sometimes the presence of a smell makes it hard for me to focus on anything else	◯	◯	◯	◯	◯
32	There are certain repetitive actions that others consider to be 'characteristic' of me (e.g. stroking my hair)	◯	◯	◯	◯	◯
33	Metaphors or ‘figures of speech’ often confuse me	◯	◯	◯	◯	◯
34	It annoys me when plans I have made are changed	◯	◯	◯	◯	◯
35	I find it difficult to make new friends	◯	◯	◯	◯	◯
36	I react poorly to unexpected loud noises	◯	◯	◯	◯	◯
37	I have difficulty understanding someone else’s point-of-view	◯	◯	◯	◯	◯
38	I like to arrange items in rows or patterns	◯	◯	◯	◯	◯
39	I try to follow certain 'rules' in order to get by in social situations	◯	◯	◯	◯	◯
40	I am sensitive to flickering lights	◯	◯	◯	◯	◯
41	I have certain habits that I find difficult to stop (e.g. biting/tearing nails, pulling strands of hair)	◯	◯	◯	◯	◯
42	I have difficulty understanding the 'unspoken rules' of social situations	◯	◯	◯	◯	◯
		Definitely Disagree	Somewhat Disagree	Neither Agree nor Disagree	Somewhat Agree	Definitely Agree

TOTAL	SOC	COM	CAM	RIG	REP	SEN
42-210	7-35	7-35	7-35	7-35	7-35	7-35

Not seeing automatically calculated totals? Make sure you haven't missed any questions. Otherwise, you may be using an incompatible PDF viewer or using an edited version of the questionnaire with the JavaScript used for the calculations removed.

REMOVE ALL DATA FROM FORM

Charts represent the distribution of CATI scores from ≈1100 individuals from the general population (see English et al. [2021] for details). The grey region shows the middle 50% of scores and the white line in the middle of the grey region is the mean.

TOTAL — Total Scale

All items. 8, 15, 19, 23, 28 reverse-scored

A very broad representation of overall autistic-like traits. 'High' total-scale scores do not necessarily mean that every subscale score is also 'high'.

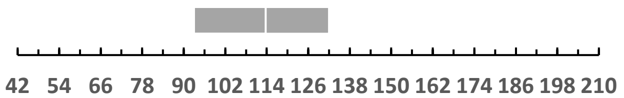

SOC — Social Interactions

Items 8, 10, 15*, 17, 28*, 30, 35 (* reversed-scored)*

Higher scores indicate greater difficulty and/or reduced preference for being involved in social situations, particularly with less-familiar individuals.

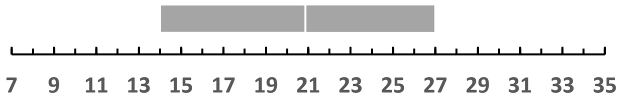

COM — Communication

Items 13, 19, 23*, 26, 33, 37, 42 (* reversed-scored)*

Higher scores indicate greater difficulty with indirect and non-verbal forms of communication.

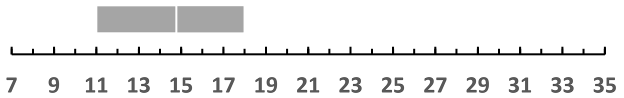

CAM — Social Camouflage

Items 3, 6, 9, 16, 22, 29, 39

Higher scores indicate increased likelihood to engage in compensatory and/or masking behaviours in social situations to 'fit in'.

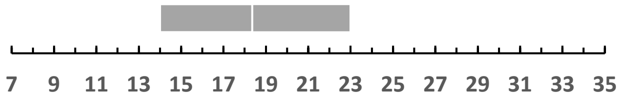

RIG — Cognitive Rigidity

Items 2, 5, 14, 21, 27, 34, 38

Higher scores indicate greater preference for 'sameness' and resistance to change (e.g. routines).

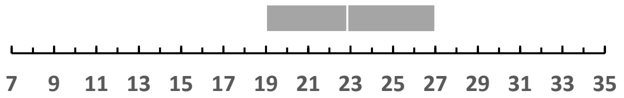

REP — Repetitive Behaviour

Items 1, 7, 12, 20, 25, 32, 41

Higher scores indicate increased prevalence of repetitive physical behaviours.

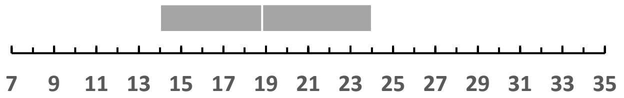

SEN — Sensory Sensitivity

Items 4, 11, 18, 24, 31, 36, 40

Higher scores indicate greater responsiveness and sensitivity to sensory stimuli.

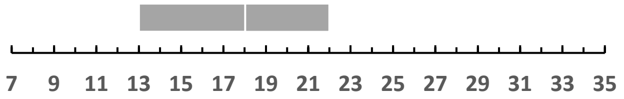

DISCLAIMER: The CATI is intended to provide a descriptive summary of traits and characteristics qualitatively similar to those found in clinical autism. It is NOT intended to be diagnostic or replace a diagnosis received by qualified clinicians using clinically-validated tools.

For more information on the development of the CATI, please see the following article:

MCW English, GE Gignac, TAW Visser, AJO Whitehouse, JT Enns, & MT Maybery (2021). **The Comprehensive Autistic Trait Inventory (CATI): Development and validation of a new measure of autistic traits in the general population**. *Molecular Autism*.

A simplified version of the CATI and scoring key can be found in the supplementary material of the above article.
