# Supplementary material for: The Comprehensive Autistic Trait Inventory (CATI): development and validation of a new measure of autistic traits in the general population
Source: Mol Autism. 2021 May 17;12:37. doi: 10.1186/s13229-021-00445-7 (PMC8130295; doi:10.1186/s13229-021-00445-7)
Supplement: Supplementary file 2 — Additional file 2. PDF versions of the CATI and scoring key. [file 13229_2021_445_MOESM2_ESM.zip › CATI - scoring key.pdf]

CATI

SCORING KEY

All items are scored 1 to 5 ('Definitely Disagree' to 'Definitely Agree') except items 8, 15, 19, 23, and 28 (highlighted below), which are reversed. Total scale score ranges from 42 – 210, whilst subscales range 7 – 35.

The six subscales are each made up of seven items. The subscale for each item is listed in the second column.

SOC = Social Interactions
COM = Communication
CAM = Social Camouflage
RIG = Cognitive Rigidity
REP = Repetitive Behaviours
SEN = Sensory Sensitivity

			Definitely Disagree	Somewhat Disagree	Neither Agree nor Disagree	Somewhat Agree	Definitely Agree
1	REP	I often find myself fiddling or playing repetitively with objects (e.g. clicking pens)	1	2	3	4	5
2	RIG	I like to stick to certain routines for every-day tasks	1	2	3	4	5
3	CAM	I expend a lot of mental energy trying to fit in with others	1	2	3	4	5
4	SEN	I am over-sensitive to bright lighting	1	2	3	4	5
5	RIG	There are certain activities that I always choose to do the same way, every time	1	2	3	4	5
6	CAM	Sometimes I watch people interacting and try to copy them when I need to socialise	1	2	3	4	5
7	REP	I often rock when sitting in a chair	1	2	3	4	5
8	SOC	I generally enjoy social events	5	4	3	2	1
9	CAM	I look for strategies and ways to appear more sociable	1	2	3	4	5
10	SOC	In social situations, I try to avoid interactions with other people	1	2	3	4	5
11	SEN	There are times when I feel that my senses are overloaded	1	2	3	4	5
12	REP	There are certain objects that I fiddle or play with that can help me calm down or collect my thoughts	1	2	3	4	5
13	COM	Reading non-verbal cues (e.g. facial expressions, body language) is difficult for me	1	2	3	4	5
14	RIG	I like my belongings to be sorted in certain ways and will spend time making sure they are that way	1	2	3	4	5
15	SOC	Social interaction is easy for me	5	4	3	2	1
16	CAM	When interacting with other people, I spend a lot of effort monitoring how I am coming across	1	2	3	4	5
17	SOC	I find social interactions stressful	1	2	3	4	5
18	SEN	I am over-sensitive to touch	1	2	3	4	5
19	COM	I can tell how people feel from their facial expressions	5	4	3	2	1
			Definitely Disagree	Somewhat Disagree	Neither Agree nor Disagree	Somewhat Agree	Definitely Agree

			Definitely Disagree	Somewhat Disagree	Neither Agree nor Disagree	Somewhat Agree	Definitely Agree
**20**	**REP**	**I have a tendency to pace or move around in a repetitive path**	**1**	**2**	**3**	**4**	**5**
**21**	**RIG**	**I feel discomfort when prevented from completing a particular routine**	**1**	**2**	**3**	**4**	**5**
**22**	**CAM**	**I rely on a set of scripts when I talk with people**	**1**	**2**	**3**	**4**	**5**
**23**	**COM**	**I find it easy to sense what someone else is feeling**	**5**	**4**	**3**	**2**	**1**
**24**	**SEN**	**I am over-sensitive to particular tastes (e.g. salty, sour, spicy, or sweet)**	**1**	**2**	**3**	**4**	**5**
**25**	**REP**	**I engage in certain repetitive actions when I feel stressed**	**1**	**2**	**3**	**4**	**5**
**26**	**COM**	**I rarely use non-verbal cues in my interactions with others**	**1**	**2**	**3**	**4**	**5**
**27**	**RIG**	**I often insist on doing things in a certain way, or re-doing things until they are 'just right'**	**1**	**2**	**3**	**4**	**5**
**28**	**SOC**	**I feel confident or capable when meeting new people**	**5**	**4**	**3**	**2**	**1**
**29**	**CAM**	**Before engaging in a social situation, I will create a script to follow where possible**	**1**	**2**	**3**	**4**	**5**
**30**	**SOC**	**Social occasions are often challenging for me**	**1**	**2**	**3**	**4**	**5**
**31**	**SEN**	**Sometimes the presence of a smell makes it hard for me to focus on anything else**	**1**	**2**	**3**	**4**	**5**
**32**	**REP**	**There are certain repetitive actions that others consider to be 'characteristic' of me (e.g. stroking my hair)**	**1**	**2**	**3**	**4**	**5**
**33**	**COM**	**Metaphors or 'figures of speech' often confuse me**	**1**	**2**	**3**	**4**	**5**
**34**	**RIG**	**It annoys me when plans I have made are changed**	**1**	**2**	**3**	**4**	**5**
**35**	**SOC**	**I find it difficult to make new friends**	**1**	**2**	**3**	**4**	**5**
**36**	**SEN**	**I react poorly to unexpected loud noises**	**1**	**2**	**3**	**4**	**5**
**37**	**COM**	**I have difficulty understanding someone else's point-of-view**	**1**	**2**	**3**	**4**	**5**
**38**	**RIG**	**I like to arrange items in rows or patterns**	**1**	**2**	**3**	**4**	**5**
**39**	**CAM**	**I try to follow certain 'rules' in order to get by in social situations**	**1**	**2**	**3**	**4**	**5**
**40**	**SEN**	**I am sensitive to flickering lights**	**1**	**2**	**3**	**4**	**5**
**41**	**REP**	**I have certain habits that I find difficult to stop (e.g. biting/tearing nails, pulling strands of hair)**	**1**	**2**	**3**	**4**	**5**
**42**	**COM**	**I have difficulty understanding the 'unspoken rules' of social situations**	**1**	**2**	**3**	**4**	**5**
			**Definitely Disagree**	**Somewhat Disagree**	**Neither Agree nor Disagree**	**Somewhat Agree**	**Definitely Agree**
