# Supplementary material for: Correction to: The Comprehensive Autistic Trait Inventory (CATI): development and validation of a new measure of autistic traits in the general population
Source: Mol Autism. 2021 Nov 8;12:70. doi: 10.1186/s13229-021-00475-1 (PMC8573992; doi:10.1186/s13229-021-00475-1)
Supplement: Supplementary file 1 — Additional file 1. Supplementary tables and figures. [file 13229_2021_475_MOESM1_ESM.docx]

# The Comprehensive Autistic Trait Inventory (CATI): Development and validation of a new measure of autistic traits in the general population

## Supplementary Material

**Supplementary Table S1.**

Extended participant descriptives for each of the three studies (including separate statistics for Study 2, with and without the additional autistic participants. “Non-binary” includes responses of “agender”, “variable”, and “gender-fluid”.

| **Study 1** |  | **Total Sample** | **Non-autistic** | **Autistic (Self-Identify)** | **Autistic (Diagnosed)** |
| --- | --- | --- | --- | --- | --- |
| Total |  | 1166 | 1119 | 30 | 17 |
| Sex | Male | 569 | 545 | 14 | 10 |
|  | Female | 581 | 559 | 16 | 6 |
|  | Intersex | 0 | 0 | 0 | 0 |
|  | Not Given | 16 | 15 | 0 | 1 |
| Gender | Man | 556 | 533 | 13 | 10 |
|  | Woman | 560 | 541 | 15 | 4 |
|  | Trans-man | 2 | 2 | 0 | 0 |
|  | Trans-woman | 3 | 3 | 0 | 0 |
|  | Non-binary | 7 | 3 | 2 | 2 |
|  | Not Given | 38 | 37 | 0 | 1 |
| Age (years) | Mean | 37.33 | 37.65 | 31.4 | 27 |
|  | SD | 12.85 | 12.89 | 9.29 | 8.15 |
|  | Range | 18-82 | 18-82 | 18-56 | 18-46 |

| **Study 2** |  | **Total Sample** | **Non-autistic** | **Autistic (Self-Identify)** | **Autistic (Diagnosed)** |
| --- | --- | --- | --- | --- | --- |
| Total |  | 1119 | 1068 | 42 | 9 |
| Sex | Male | 557 | 530 | 24 | 3 |
|  | Female | 552 | 529 | 18 | 5 |
|  | Intersex | 0 | 0 | 0 | 0 |
|  | Not Given | 10 | 9 | 0 | 1 |
| Gender | Man | 540 | 515 | 22 | 3 |
|  | Woman | 529 | 510 | 15 | 4 |
|  | Trans-man | 4 | 2 | 2 | 0 |
|  | Trans-woman | 5 | 5 | 0 | 0 |
|  | Non-binary | 5 | 2 | 1 | 2 |
|  | Not Given | 36 | 34 | 2 | 0 |
| Age (years) | Mean | 37.41 | 37.38 | 38.19 | 37.67 |
|  | SD | 12.59 | 12.56 | 13.64 | 12.77 |
|  | Range | 18-75 | 18-74 | 18-75 | 20-52 |

| **Study 2 (expanded sample for regressions)** | | **Total Sample** | **Non-autistic** | **Autistic (Self-Identify)** | **Autistic (Diagnosed)** |
| --- | --- | --- | --- | --- | --- |
| Total |  | 1209 | 1076 | 77 | 56 |
| Sex | Male | 600 | 535 | 42 | 23 |
|  | Female | 595 | 532 | 35 | 28 |
|  | Intersex | 1 | 0 | 0 | 1 |
|  | Not Given | 13 | 9 | 0 | 4 |
| Gender | Man | 582 | 520 | 39 | 23 |
|  | Woman | 562 | 513 | 29 | 20 |
|  | Trans-man | 4 | 2 | 2 | 0 |
|  | Trans-woman | 5 | 5 | 0 | 0 |
|  | Non-binary | 14 | 2 | 4 | 8 |
|  | Not Given | 42 | 34 | 3 | 5 |
| Age (years) | Mean | 36.88 | 37.37 | 35.42 | 29.3 |
|  | SD | 12.54 | 12.56 | 12.13 | 10.22 |
|  | Range | 18-75 | 18-74 | 18-75 | 18-52 |

| **Study 3** | | **Total Sample** | **Non-autistic** | **Autistic (Self-Identify)** | **Autistic (Diagnosed)** |
| --- | --- | --- | --- | --- | --- |
| Total |  | 202 | 195 | 4 | 3 |
| Sex | Male | 103 | 100 | 2 | 1 |
|  | Female | 99 | 95 | 2 | 2 |
|  | Intersex | 0 | 0 | 0 | 0 |
|  | Not Given | 0 | 0 | 0 | 0 |
| Gender | Man | 99 | 96 | 2 | 1 |
|  | Woman | 96 | 92 | 2 | 2 |
|  | Trans-man | 0 | 0 | 0 | 0 |
|  | Trans-woman | 0 | 0 | 0 | 0 |
|  | Non-binary | 3 | 3 | 0 | 0 |
|  | Not Given | 4 | 4 | 0 | 0 |
| Age (years) | Mean | 33.71 | 33.75 | 30 | 36.33 |
|  | SD | 11.81 | 11.67 | 11.78 | 23.44 |
|  | Range | 18-71 | 18-71 | 18-42 | 19-63 |

**Supplementary Table S2.**

Results from analyses on the first exploratory sample and 107-item pilot version of the CATI, including factor analysis item loadings, and outcomes of t-tests comparing item scores between non-autistic and autistic participants (ASD: diagnosed and self-identifying combined). Shaded items were selected for the 42-item final version of the CATI with explanatory notes outlining why otherwise high-loading items were not included. Loadings ≥ .30 are bolded. Reverse-keyed items marked with a *.

|  | **Loading** | | | | | | **ASD discrimination** | | **Comment** |
| --- | --- | --- | --- | --- | --- | --- | --- | --- | --- |
| **Item** | **SOC** | **COM** | **SEN** | **REP** | **CAM** | **RIG** | ***p*** | ***d*** |  |
| **Social Interactions (SOC)** |  |  |  |  |  |  |  |  |  |
| I generally enjoy social events* | **.79** | .00 | .11 | -.08 | .03 | .01 | <.001 | 0.73 | Item retained |
| Social interaction is easy for me* | **.79** | .09 | -.01 | -.02 | .18 | .01 | <.001 | 0.93 | Item retained |
| I find social interactions stressful | **.72** | -.06 | .14 | .03 | .27 | -.02 | <.001 | 0.82 | Item retained |
| I am confident and capable when meeting new people* | **.71** | .10 | -.06 | .05 | .14 | .02 | <.001 | 0.68 | Item retained |
| Social occasions are often challenging for me | **.69** | .02 | .11 | -.01 | .29 | .02 | <.001 | 0.92 | Item retained |
| I find it difficult to make new friends | **.65** | .12 | -.05 | -.07 | .25 | .04 | <.001 | 0.71 | Item retained |
| In social situations, I try to avoid interactions with other people | **.65** | .06 | .01 | .06 | .26 | .02 | <.001 | 0.86 | Item retained |
| Maintaining friendships is easy for me* | **.61** | .15 | .02 | -.01 | .15 | -.03 | <.001 | 0.65 |  |
| I feel uncomfortable participating in idle social chit-chat | **.60** | .09 | .00 | .06 | .17 | .05 | <.001 | 0.73 |  |
| I usually feel exhausted after social events | **.58** | -.18 | **.31** | .06 | .18 | -.01 | <.001 | 0.62 |  |
| I can be myself in social situations* | **.58** | .08 | .04 | -.13 | **.40** | -.05 | <.001 | 1.11 |  |
| I am often awkward in my interactions with others | **.57** | .08 | .00 | .08 | **.37** | .02 | <.001 | 0.97 |  |
| I am skilled in working as part of a group* | **.56** | .24 | .01 | .06 | -.04 | -.03 | <.001 | 1.04 |  |
| I don’t see the point in idle social chit-chat or small-talk | **.52** | .12 | .06 | .01 | .04 | .07 | <.001 | 0.64 |  |
| I need to spend time by myself to cope following social interactions | **.51** | -.13 | .27 | .08 | .22 | .05 | <.001 | 0.78 |  |
| I would prefer to have my birthday by myself than spend it with others | **.49** | .07 | .02 | -.06 | .08 | .11 | <.001 | 0.51 |  |
| I am skilful in dealing with others on difficult issues* | **.49** | **.39** | -.10 | .06 | -.06 | .01 | <.001 | 0.74 |  |
| I am good at managing unexpected changes* | **.43** | .15 | .10 | .10 | -.08 | .16 | <.001 | 0.82 |  |
| I am good at multi-tasking* | **.42** | .22 | .04 | .24 | -.25 | -.03 | <.001 | 0.69 |  |
| Friends sometimes seek my advice if they are having problems in their relationships* | **.42** | **.40** | -.20 | -.11 | -.06 | .08 | .050 | 0.31 |  |
| My free time is occupied by a wide range of activities* | **.41** | .16 | -.16 | -.11 | .02 | .17 | .010 | 0.40 |  |
| It is difficult for me to relate to other people | **.40** | **.36** | .08 | .01 | .17 | -.01 | <.001 | 1.05 |  |
| I don't like it when I'm on my own* | **.39** | -.16 | -.03 | -.22 | -.07 | .12 | .800 | 0.04 |  |
| How I appear to other people is probably quite similar to how I really am* | **.38** | .03 | .03 | .01 | **.35** | -.13 | <.001 | 0.65 |  |
| I can generally focus on more than one thing at a time* | **.37** | .28 | .06 | .21 | -.25 | .01 | .010 | 0.52 |  |
| I am able to communicate my needs to others* | **.35** | **.32** | -.02 | .07 | .14 | -.10 | <.001 | 0.73 |  |
| I have a broad range of interests and activities* | **.32** | .29 | -.14 | -.13 | .03 | .15 | .010 | 0.48 |  |
| **Communication (COM)** |  |  |  |  |  |  |  |  |  |
| Reading non-verbal cues (e.g. facial expressions, body language) is difficult for me | -.02 | **.78** | -.01 | .07 | .09 | -.04 | <.001 | 1.46 | Item retained |
| I can tell how people feel from their facial expressions* | .00 | **.74** | -.05 | -.02 | -.11 | -.03 | <.001 | 1.30 | Item retained |
| I find it easy to sense what someone else is feeling* | .10 | **.70** | -.11 | -.06 | -.07 | .02 | <.001 | 1.07 | Item retained |
| Metaphors or ‘figures of speech’ often confuse me | -.26 | **.64** | .14 | .07 | .12 | .02 | <.001 | 0.73 | Item retained |
| I rarely use non-verbal cues in my interactions with others | -.11 | **.62** | .01 | -.07 | .03 | .13 | <.001 | 0.82 | Item retained |
| I have difficulty understanding the 'unspoken rules' of social situations | .14 | **.59** | .02 | .08 | .23 | -.05 | <.001 | 1.12 | Item retained |
| I have difficulty understanding someone else’s point-of-view | .02 | **.59** | .05 | .06 | .07 | .04 | <.001 | 0.99 | Item retained |
| I rarely misinterpret social cues* | .15 | **.54** | -.09 | .09 | .04 | -.03 | <.001 | 1.00 |  |
| I have trouble understanding what others want from me if they do not use plain, direct language | .07 | **.52** | .12 | .09 | .11 | .09 | <.001 | 1.07 |  |
| I have difficulty understanding what other people want from me | .11 | **.51** | .08 | .08 | .26 | -.04 | <.001 | 1.14 |  |
| I find it easy to change my behaviour to suit different social situations* | .28 | **.46** | .07 | -.04 | -.12 | -.01 | <.001 | 0.90 |  |
| I like the feel of different textures and surfaces [TOUCH] | -.07 | **-.35** | .10 | .17 | .11 | .02 | .710 | 0.06 |  |
| The 'back-and-forth' turn-taking in conversations is difficult to me | **.31** | **.32** | .04 | .14 | .25 | -.02 | <.001 | 0.96 |  |
| I find it hard to switch from one activity to another | .22 | .29 | .12 | .19 | .02 | .09 | <.001 | 0.97 |  |
| I like the physical sensation of being hugged [TOUCH]* | .24 | .28 | .15 | -.23 | -.04 | .02 | .090 | 0.29 |  |
| I prefer foods with complex and intense flavours [TASTE]* | .05 | .27 | -.01 | -.23 | -.03 | .11 | .640 | 0.08 |  |
| **Sensory Sensitivity** |  |  |  |  |  |  |  |  |  |
| I react poorly to unexpected loud noises [SOUND] | .14 | .01 | **.66** | .02 | -.02 | .03 | <.001 | 0.75 | Highest loading SOUND item |
| I am comfortable with the sounds made by fireworks [SOUND]* | .05 | .17 | **.63** | -.19 | -.06 | -.06 | <.001 | 0.44 |  |
| I tire fast in noisy environments [SOUND] | **.35** | -.05 | **.63** | -.04 | -.03 | -.01 | <.001 | 0.77 |  |
| I am not bothered by being in noisy environments (e.g. shopping centres, cinemas) [SOUND]* | **.33** | .06 | **.62** | -.07 | -.16 | -.04 | <.001 | 0.85 |  |
| I am sensitive to flickering lights [SIGHT] | .08 | -.03 | **.61** | .01 | .04 | -.01 | <.001 | 0.54 | Highest loading SIGHT item |
| Loud noises distract me from my thoughts [SOUND] | .29 | -.09 | **.59** | .08 | -.13 | .02 | .010 | 0.42 |  |
| I cannot tolerate certain everyday noises such as lawnmowers [SOUND] | .07 | .10 | **.53** | .04 | -.03 | .01 | <.001 | 0.72 |  |
| I am over-sensitive to bright lighting [SIGHT] | -.07 | -.09 | **.52** | .11 | .09 | .13 | <.001 | 0.90 | Second highest loading SIGHT item |
| I am over-sensitive to touch [TOUCH] | .07 | .16 | **.51** | .00 | .19 | .03 | <.001 | 1.20 | Highest loading TOUCH item |
| There are times when I feel that my senses are overloaded [GENERAL] | .15 | -.08 | **.49** | .27 | .07 | -.01 | <.001 | 0.99 | Highest loading GENERAL item |
| I find many kinds of touch unpleasant [TOUCH] | .07 | .25 | **.48** | -.09 | .16 | .04 | <.001 | 1.07 |  |
| I am over-sensitive to particular tastes (e.g. salty, sour, spicy, or sweet) [TASTE] | -.16 | .28 | **.47** | .03 | .08 | .05 | .010 | 0.48 | Highest loading TASTE item |
| I feel sick when I smell certain odours [SMELL] | -.11 | .00 | **.47** | .05 | .05 | .06 | .990 | 0.00 | Highest loading SMELL item, but did not distinguish ASD & non-ASD |
| Sometimes the presence of a smell makes it hard for me to focus on anything else [SMELL] | -.11 | .09 | **.46** | .10 | .03 | .12 | .160 | 0.24 | Second-highest loading SMELL item |
| I find the texture of certain foods unpleasant [TOUCH] | -.09 | .09 | **.39** | .04 | -.02 | .14 | .020 | 0.36 |  |
| I avoid food with certain textures [TOUCH] | -.08 | .17 | **.38** | .08 | -.03 | .12 | <.001 | 0.48 |  |
| I prefer not to be touched (e.g. shaking hands) [TOUCH] | **.31** | .17 | **.38** | -.17 | .14 | .01 | <.001 | 0.89 |  |
| I am over-sensitive to being tickled [TOUCH] | -.06 | .07 | **.38** | -.03 | .18 | -.01 | <.001 | 0.60 |  |
| I don’t like the feel of clothing tags [TOUCH] | .11 | -.05 | **.35** | .11 | -.09 | .11 | <.001 | 0.48 |  |
| If someone is trying to speak to me, I must finish what I am working on before I can listen to them | .11 | .18 | .24 | .18 | .01 | .16 | <.001 | 0.77 |  |
| I tend to smell new foods before I taste them [SMELL] | -.09 | -.07 | .21 | .07 | .04 | .13 | .600 | 0.08 |  |
| **Repetitive Behaviour (REP)** |  |  |  |  |  |  |  |  |  |
| I often find myself fiddling or playing repetitively with objects (e.g. clicking pens) | .11 | -.04 | .00 | **.80** | -.02 | .00 | <.001 | 0.75 | Item retained |
| I often tap my feet when I am sitting down | .08 | .02 | -.10 | **.73** | -.06 | -.03 | <.001 | 0.54 | Skipped to select another item with better ASD discrimination |
| There are certain objects that I fiddle or play with that can help me calm down or collect my thoughts | -.02 | .08 | .08 | **.69** | -.06 | .06 | <.001 | 0.76 | Item retained |
| I often rock when sitting in a chair | -.01 | .08 | -.01 | **.66** | -.01 | -.02 | <.001 | 0.66 | Item retained |
| I have a tendency to pace or move around in a repetitive path | .05 | .11 | .04 | **.61** | .02 | .08 | <.001 | 0.80 | Item retained |
| I engage in certain repetitive actions when I feel stressed | .10 | .00 | .02 | **.57** | .06 | .21 | <.001 | 0.74 | Item retained |
| There are certain repetitive actions that others consider to be 'characteristic' of me (e.g. stroking my hair) | -.07 | .07 | -.04 | **.57** | .10 | .16 | <.001 | 0.87 | Item retained |
| I often hold a small object in my hands because I like the way it feels [TOUCH] | -.14 | .08 | .18 | **.54** | .08 | .02 | <.001 | 0.63 | Did not mesh well with other items |
| I have certain habits that I find difficult to stop (e.g. biting/tearing nails, pulling strands of hair) | .09 | -.02 | -.03 | **.53** | .05 | .09 | <.001 | 0.70 | Item retained |
| I feel calmed by making repetitive noises to myself (e.g. humming) | -.08 | .14 | .03 | **.50** | .11 | .09 | <.001 | 0.92 |  |
| My thoughts often jump from topic to topic* | -.05 | .12 | -.12 | **-.42** | -.15 | .10 | <.001 | 0.55 |  |
| I am sometimes so intensely focused on something that I fail to take care of my responsibilities | .10 | .16 | .11 | **.39** | .09 | .00 | <.001 | 0.99 |  |
| I prefer to wear headphones to block out noises [SOUND] | .13 | .01 | .17 | **.34** | .06 | .02 | <.001 | 0.71 |  |
| I prefer to discuss my interests or hobbies to the exclusion of other conversational topics | -.04 | **.30** | -.07 | **.31** | .12 | .12 | <.001 | 0.89 |  |
| I have certain catchphrases that I often use when speaking | -.16 | .03 | .00 | **.31** | .23 | .15 | <.001 | 0.45 |  |
| My intense interests sometimes mean I neglect things that other people think important | .15 | .18 | .08 | .29 | .10 | .08 | <.001 | 1.08 |  |
| I own certain objects purely for how they feel [TOUCH] | -.25 | .06 | .26 | .26 | .16 | .06 | <.001 | 0.46 |  |
| **Social Camouflage (CAM)** |  |  |  |  |  |  |  |  |  |
| I expend a lot of mental energy trying to fit in with others | .21 | -.03 | .04 | .11 | **.65** | -.04 | <.001 | 0.96 | Item retained |
| When socialising, I feel like I am pretending to be someone else to fit in | **.38** | -.01 | -.02 | .01 | **.65** | -.03 | <.001 | 0.78 | Cross-loaded on Social factor |
| Sometimes I watch people interacting and try to copy them when I need to socialise | .03 | .14 | -.01 | .08 | **.65** | .05 | <.001 | 0.91 | Item retained |
| I look for strategies and ways to appear more sociable | -.04 | -.02 | -.02 | .07 | **.63** | .11 | <.001 | 0.55 | Item retained |
| In social situations, I feel I am putting on an 'act' to fit in | **.46** | -.07 | -.04 | -.01 | **.62** | .01 | <.001 | 0.58 | Cross-loaded on Social factor |
| I try to follow certain 'rules' in order to get by in social situations | -.04 | .09 | .05 | -.13 | **.57** | .28 | <.001 | 0.75 | Item retained |
| When interacting with other people, I spend a lot of effort monitoring how I am coming across | .14 | -.12 | .08 | .14 | **.56** | .09 | <.001 | 0.57 | Item retained |
| I rely on a set of scripts when I talk with people | .09 | .27 | -.01 | .01 | **.53** | .06 | <.001 | 0.82 | Item retained |
| Before engaging in a social situation, I will create a script to follow where possible | .13 | .18 | .02 | .06 | **.52** | .06 | <.001 | 0.74 | Item retained |
| I try to hide the 'real me' from others | **.38** | .01 | .00 | -.04 | **.51** | .00 | <.001 | 0.62 |  |
| I often check with someone else to see if I interpreted a social situation correctly | -.09 | .15 | .18 | .18 | **.38** | .01 | <.001 | 0.87 |  |
| It is difficult for me to know how to behave when interacting with other people | **.30** | **.34** | .03 | .09 | **.36** | -.03 | <.001 | 1.12 |  |
| I wish I had more close friends* | -.09 | -.01 | .08 | -.05 | **-.31** | -.05 | .020 | 0.36 |  |
| I have to make a conscious effort to remember to look at someone when speaking | .11 | .26 | .03 | .17 | .26 | .07 | <.001 | 1.02 |  |
| **Cognitive Rigidity (RIG)** |  |  |  |  |  |  |  |  |  |
| I like to stick to certain routines for every-day tasks | .20 | .00 | .00 | -.12 | .00 | **.72** | <.001 | 0.52 | Item retained |
| There are certain activities that I always choose to do the same way, every time | .18 | -.04 | -.09 | .03 | -.03 | **.71** | <.001 | 0.59 | Item retained |
| I like my belongings to be sorted in certain ways and will spend time making sure they are that way | -.09 | .01 | .10 | .04 | .03 | **.62** | <.001 | 0.51 | Item retained |
| I often insist on doing things in a certain way, or re-doing things until they are ‘just right’ | -.02 | .05 | .07 | .10 | .03 | **.58** | <.001 | 0.55 | Item retained |
| I feel discomfort when prevented from completing a particular routine | .13 | .01 | .12 | .09 | .04 | **.57** | <.001 | 0.69 | Item retained |
| I like to arrange items in rows or patterns | -.09 | .08 | .12 | .19 | .04 | **.45** | <.001 | 0.56 | Item retained |
| It annoys me when plans I have made are changed | .21 | -.05 | .11 | .08 | .00 | **.40** | <.001 | 0.58 | Item retained |
| I like to spend a lot of my time on a small number of interests | .26 | .01 | -.01 | .08 | -.03 | **.38** | <.001 | 0.69 |  |
| I tend to wear the same style of clothes from day to day | **.32** | -.04 | -.04 | -.01 | -.02 | **.36** | <.001 | 0.65 |  |
| I get agitated if I can’t spend time on the things that interest me | .24 | -.04 | .11 | .26 | -.01 | **.31** | <.001 | 0.64 |  |
| I am not bothered if I take a wrong turn walking or driving somewhere* | .18 | .03 | .18 | -.10 | .05 | .21 | .010 | 0.40 |  |

**Supplementary Table S3.**

Sensitivity and specificity for the CATI total-scale and subscales. Cut-off scores indicate the lowest score for identifying autism (inclusive) that resulted in the highest sum combination of sensitivity (correctly identifying autistic participants) and specificity (correctly identifying non-autistic participants).

|  | **Cut-off for ASD** | **Sensitivity (A)** | **Specificity (B)** | **Average (A+B)** | **Youden’s Index** |
| --- | --- | --- | --- | --- | --- |
| **CATI** |  |  |  |  |  |
| *Social Interactions* | 29 | 65.41% | 81.51% | 73.46% | .47 |
| *Sensory Sensitivity* | 24 | 69.92% | 80.95% | 75.44% | .51 |
| *Repetitive Behaviour* | 24 | 74.44% | 75.00% | 74.72% | .49 |
| *Communication* | 18 | 80.45% | 75.37% | 77.91% | .56 |
| *Cognitive Rigidity* | 26 | 74.44% | 68.31% | 71.37% | .43 |
| *Social Camouflage* | 25 | 66.92% | 84.01% | 75.47% | .51 |
| ***Average of Factors*** |  | **72.93%** | **76.23%** | **74.58%** | **.50** |
| ***Total Scale*** | **134** | **82.71%** | **79.00%** | **80.85%** | **.62** |
|  |  |  |  |  |  |
| **AQ** |  |  |  |  |  |
| *Social Skill* | 28 | 72.93% | 77.32% | 75.13% | .50 |
| *Attention Switching* | 30 | 73.68% | 82.25% | 77.97% | .56 |
| *Attention to Detail* | 28 | 53.38% | 68.87% | 61.12% | .22 |
| *Communication* | 24 | 84.21% | 72.96% | 78.58% | .57 |
| *Imagination* | 24 | 50.38% | 78.25% | 64.31% | .29 |
| ***Average of Factors*** |  | **66.92%** | **75.93%** | **71.42%** | **.43** |
| ***Total Scale*** | **132** | **72.93%** | **86.06%** | **79.50%** | **.59** |
|  |  |  |  |  |  |
| **BAPQ** |  |  |  |  |  |
| *Aloof Personality* | 49 | 66.92% | 76.86% | 71.89% | .44 |
| *Pragmatic Language* | 43 | 63.91% | 86.80% | 75.36% | .51 |
| *Rigid Personality* | 47 | 69.92% | 77.23% | 73.58% | .47 |
| ***Average of Factors*** |  | **66.92%** | **80.30%** | **73.61%** | **.47** |
| ***Total Scale*** | **134** | **75.19%** | **80.86%** | **78.02%** | **.56** |

**Supplementary Table S4.**

Model summaries of several logistic regression analyses predicting autistic status using the CATI, AQ and/or BAPQ.

|  |  |  |  |  |  | **Model Difference** | | |
| --- | --- | --- | --- | --- | --- | --- | --- | --- |
| **First Step** | **Second Step** | ***df*** | **χ^2^** | ***p*** | **Nagelkerke’s pseudo *R*^2^** | **Δ χ^2^** | **Δ *R*^2^** | ***p*** |
| CATI | - | 6 | 332.95 | < .001 | .482 | - | - | - |
| CATI | AQ | 11 | 352.20 | < .001 | .506 | 22.86 | .024 | < .001 |
|  |  |  |  |  |  |  |  |  |
| AQ | - | 5 | 305.84 | < .001 | .447 | - | - | - |
| AQ | CATI | 11 | 352.20 | < .001 | .506 | 46.36 | .059 | < .001 |
|  |  |  |  |  |  |  |  |  |
| CATI | - | 6 | 332.95 | < .001 | .482 | - | - | - |
| CATI | BAPQ | 9 | 335.52 | < .001 | .485 | 2.56 | .007 | .46 |
|  |  |  |  |  |  |  |  |  |
| BAPQ | - | 3 | 253.46 | < .001 | .378 | - | - | - |
| BAPQ | CATI | 9 | 335.52 | < .001 | .485 | 82.06 | .107 | < .001 |


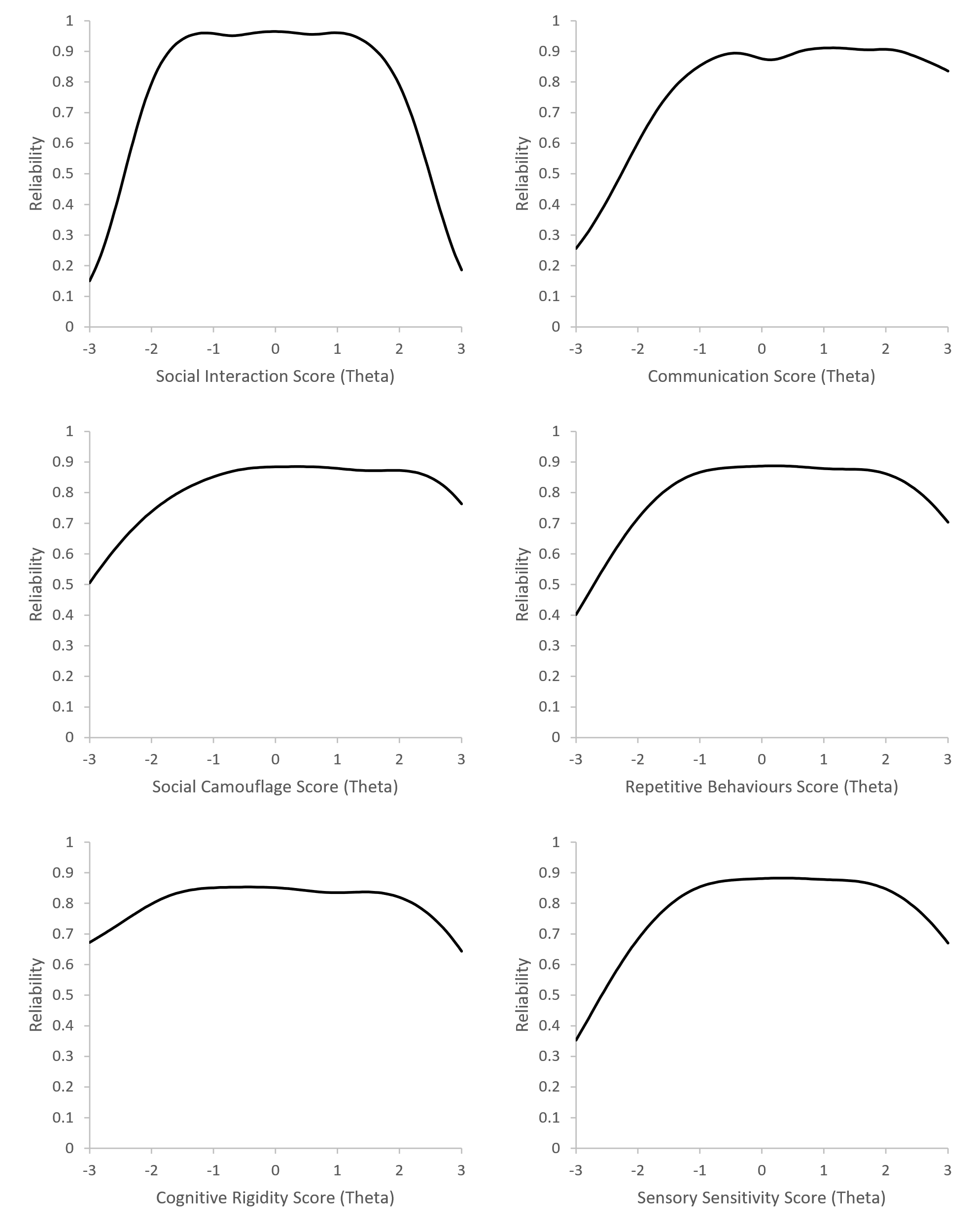


**Figure S1.** Total information curves (transformed to reliability) for the CATI subscales. X-axis represents measurement of the latent trait in standard deviation units (theta), with higher values indicative of higher levels of autistic traits.
